# Causal relationship between Alzheimer’s disease and cardiovascular disease: a bidirectional Mendelian randomization analysis

**DOI:** 10.18632/aging.205013

**Published:** 2023-09-02

**Authors:** Fengjun Zhang, Dexian Xian, Junchen Feng, Luning Ning, Tianshou Jiang, Wenchang Xu, Yuan Liu, Qiong Zhao, Min Peng

**Affiliations:** 1 College of Acupuncture and Massage, Shandong University of Traditional Chinese Medicine, Jinan, China; 2 College of Traditional Chinese Medicine, Shandong University of Traditional Chinese Medicine, Jinan, China; 3 Department of Cardiovascular Medicine, Lacey City Hospital, Qingdao, China; 4 Department of Traditional Chinese Medicine, Shandong Provincial Hospital Affiliated to Shandong First Medical University, Jinan, Shandong, China

**Keywords:** Alzheimer's disease (AD), cardiovascular disease (CVD), causality, bidirectional mendelian randomization (MR) study, genome-wide association study (GWAS)

## Abstract

Observational studies suggest that cardiovascular disease (CVD) increases the risk of developing Alzheimer’s disease (AD). However, the causal relationship between the two is not clear. This study applied a two-sample bidirectional Mendelian randomization method to explore the causal relationship between CVD and AD. Genome-wide association study (GWAS) data from 46 datasets of European populations (21,982 cases of AD and 41,944 controls) were utilized to obtain genetic instrumental variables for AD. In addition, genetic instrumental variables for atrial fibrillation (AF), heart failure (HF), myocardial infarction (MI), coronary heart disease (CHD), angina pectoris (AP), and ischemic stroke (IS) (including large-artery atherosclerotic stroke [LAS] and cardioembolic stroke [CES]) were selected from GWAS data of European populations (*P* < 5E-8). The inverse variance weighting method was employed as the major Mendelian randomization analysis method. Genetically predicted AD odds ratios (OR) (1.06) (95% CI: 1.02–1.10, *P* = 0.003) were linked to higher AP analysis. A higher genetically predicted OR for CES (0.9) (95% CI 0.82–0.99, *P* = 0.02) was linked to a decreased AD risk. This Mendelian randomized study identified AD as a risk factor for AP. In addition, CES was related to a reduced incidence of AD. Therefore, these modifiable risk factors are crucial targets for preventing and treating AD.

## INTRODUCTION

Alzheimer’s disease (AD), a primary neurodegenerative disease clinically characterized by insidious onset and progressive cognitive impairment, is one of the most common subtypes of dementia, and commonly affects people over 65 years of age [1, 2]. According to Nichols et al., the global prevalence of dementia is predicted to rise from 57.4 million cases in 2019 to 83.2 million cases in 2030, and by 2050, an estimated 152.8 million people will have dementia, with a higher prevalence in women than men and heterogeneity in the geographical distribution of incidence [3]. The incidence of dementia increases the social and economic burden [1]. AD is a complex multifactorial disease, and there is still no cure for AD. Therefore, the identification and early control of risk factors are important measures to prevent the onset and progression of AD.

Cardiovascular disease (CVD) is a collection of heart and vascular diseases consisting of ischemic heart diseases (IHD), such as myocardial infarction (MI), atrial fibrillation (AF), and heart failure (HF), and ischemic stroke (IS) [4]. Currently, CVDs are the major cause of disability and mortality around the globe, burdening individuals and society significantly. The etiology of CVD is complex and is jointly influenced by cardiometabolic, genetic, lifestyle, environmental and social risk factors.

In recent years, there has been a growing interest in research concerning the association between cardiovascular diseases and Alzheimer’s disease, as well as the management of risk factors [5–7]. Compelling evidence suggests that cardiovascular diseases contribute to the progression of Alzheimer’s disease, with the condition being more frequently observed in patients suffering from cardiovascular ailments than in the general population [8–10]. However, the common risk factors of both make the study of causality between the two complicated and controversial [11]. Observational evidence indicates a link between the incidence of atrial fibrillation and the risk of dementia, with the use of oral anticoagulants associated with a decreased risk of dementia [12]. However, a previous Mendelian randomization study found no causal relationship between genetically predicted atrial fibrillation and Alzheimer’s disease [13]. Compared to subjects without AD, those with the disease have reduced mitral valve flow efficiency during diastolic filling and impaired consolidated diastolic function and vortex formation time [14]. In addition, many studies have not adequately adjusted for confounding factors, leading to spurious associations. Thus, current studies on the connection between dementia and CVD have led to inadequate and indefinite conclusions.

However, it is not possible to determine the sequence of CVD and AD due to the limitations of follow-up time and the number of people in traditional observational studies. Moreover, both usually share common risk factors such as diabetes, obesity, hypertension, metabolic syndrome, atherosclerosis, smoking, oxidative stress, inflammation, and APOE polymorphisms [15, 16].

In Mendelian randomization (MR), a new approach to epidemiological studies, genetic variants were used as instrumental variables (IVs) to infer causal associations among exposure factors and outcomes [17, 18]. Furthermore, the IVs used in MR analysis are random assignments of genes at some point in meiosis, leading to a random dispersal of genetic variants in the population [17]. Thus, MR analyses can avoid the interference of traditional confounders [19] to a large extent and conform to the natural causal order [20, 21]. In addition, genome-wide association studies (GWAS) are being developed, and independent GWAS databases can provide reliable IVs for MR analysis. A bidirectional MR analysis was performed in this investigation to explore the causal connection between AD and CVD.

## MATERIALS AND METHODS

### Research design

**Figure 1:**
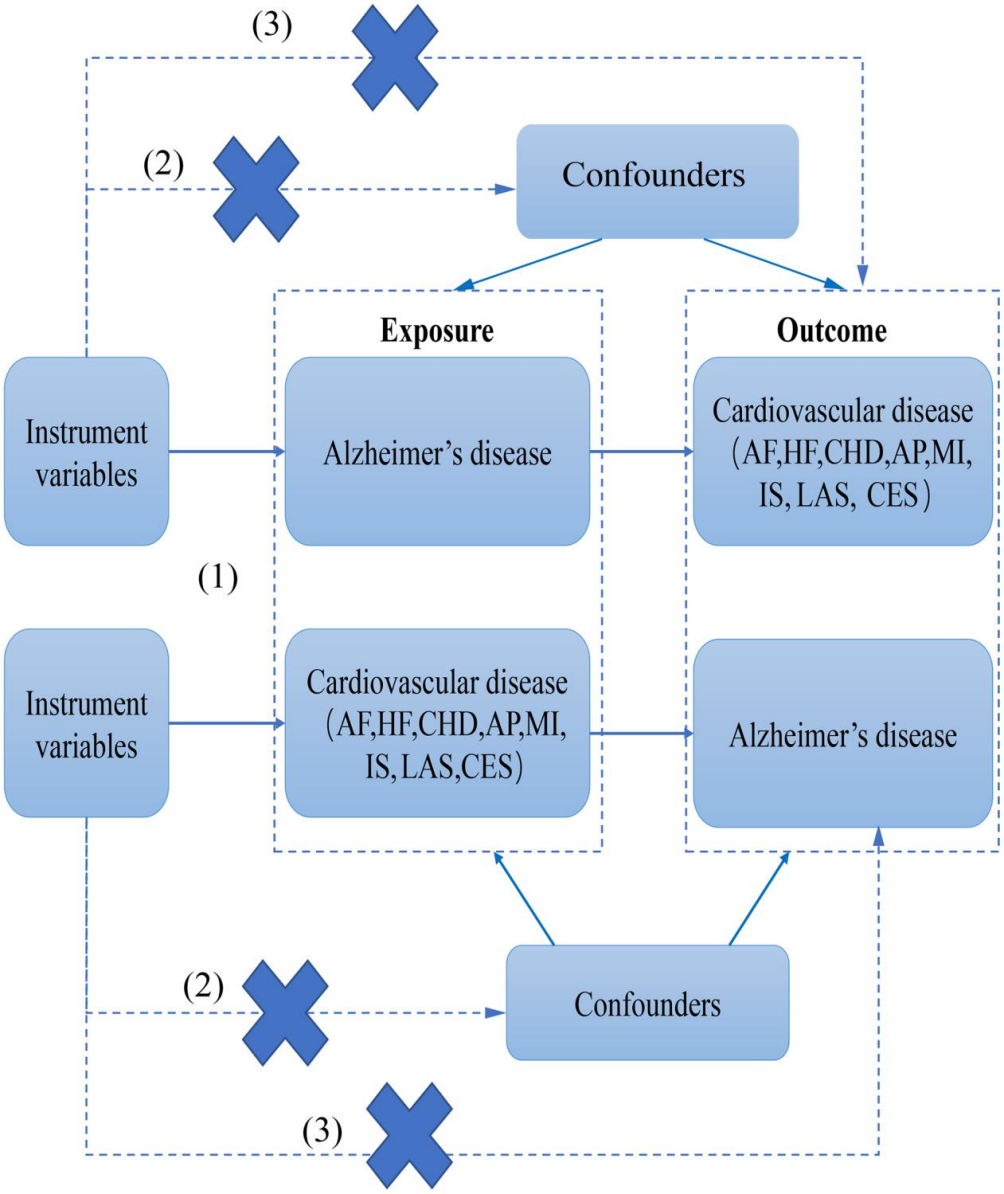
The flow chart of this study.

In this study, AD was used as “exposure” and atrial fibrillation (AF), heart failure (HF), myocardial infarction (MI), coronary heart disease (CHD), Angina pectoris (AP), and ischemic stroke (IS) (including large-artery atherosclerotic stroke (LAS), and cardioembolic stroke (CES)) as “outcome”. Subsequently, IVs for bidirectional Mendelian randomization analysis were screened for Mendelian randomization analysis. Heterogeneity was assessed using Cochran’s Q analysis. In the end, the reliability of the causal relationship was verified by performing sensitivity analyses (horizontal pleiotropy analysis and “leave-one-out” analysis). However, reverse MR was also performed to determine the sequence of CVD and AD. The following three key assumptions need to be met for MR studies: (1) association: genetic variants/IVs should be strongly linked with exposure factors; (2) independence assumption: genetic variants/IVs are independent of any confounding factors affecting exposure factors and outcome; and (3) exclusivity assumption: IVs can affect the results only through exposure and not other pathways. The present study used bidirectional Mendelian randomization to assess the causal correlation between AD and CVD (Figure 1).

### Data sources

The GWAS of AD conducted by Kunkle et al. [22] was utilized to extract pooled data for AD, including 21,982 cases of AD and 41,944 controls. The case data for the study were obtained from four separate consortia: Alzheimer Disease Genetics Consortium (ADGC), European AD Initiative (EADI), Cohorts for Heart and Aging Research in Genomic Epidemiology Consortium (CHARGE), and Genetic and Environmental Risk in AD/Defining Genetic, Polygenic and Environmental Risk for AD Consortium (GERAD/PERADES). In the second phase, the study included 8,362 cases and 10,483 controls, whereas the third phase comprised 4,930 Alzheimer’s disease cases and 6,736 controls. In both phases, clinical diagnosis of Alzheimer’s disease was based on the DSM-III-R and NINCDS-ADRDA criteria. The control group was defined as participants who did not meet the DSM-III-R criteria for dementia and had intact cognitive function (with an MMSE score greater than 25).

Pooled data for AF were obtained from a meta-analysis of GWAS studies by Roselli C et al. [23] of over 500,000 people, including 65,446 cases of AF and 522,744 controls, which was coded as follows: (1) Non-cancer illness code, self-reported (1471, 1483), (2) Operation code (1524), (3) Diagnoses – main/secondary ICD10 (I48, I48.0–4, I48.9), (4) Underlying (primary/secondary) cause of death: ICD10 (I48, I48.0–4, I48.9) (5) Diagnoses – main/secondary ICD9 (4273), (6) Operative procedures – main/secondary OPCS (K57.1, K62.1–4). Pooled data for HF were obtained from GWAS by Shah S et al. [24], enlisting 47,309 cases and 930,014 controls, and pooled statistics for CHD were obtained from CARDIoGRAM, including 22,233 cases and 64,762 controls [25]. The FinnGen dataset (https://www.finngen.fi/) was used to extract data for MI, enlisting 11,622 cases and 187,840 controls. Pooled data for AP obtained from the FinnGen dataset (https://www.finngen.fi/) included 18,168 cases and 187,840 controls. Pooled data set for IS was obtained from MEGASTROKE [26], including 34,217 IS cases and 406,111 controls. In addition, two subtypes were selected: large-artery atherosclerotic stroke (LAS) (4,373 cases) and cardioembolic stroke (CES) (7,193 cases) [26]. Table 1 shows an overview of the demographic data and GWAS involved in this study.

**Table 1:** Data description of Alzheimer’s diseases and cardiovascular disease.

**Traits**	**Data source**	**PMID**	**Year**	**Sample size (cases/controls)**	**Adjustments**	**GWAS ID**
Alzheimer’s diseases	Kunkle BW	30820047	2019	21,982/41944	Age, sex, PC, APOE and sequencing center adjusted	ieu-b-2
Atrial fibrillation	Roselli C	29892015	2018	65446/522744	Age, sex, height, body mass index (BMI), smoking, hypertension, heart failure, stroke, mitral regurgitation, bradyarrhythmia, peripheral vascular disease (PVD), hypercholesterolemia, coronary artery disease (CAD), and type II diabetes	NA
Heart failure	Shah S	31919418	2020	47309/930014	Age and sex	ebi-a-GCST009541
Coronary heart disease	Schunkert H	21378990	2011	22233/64762	Age and sex	ieu-a-8
Angina pectoris	FinnGen	NA	2021	18168/187840	Age, sex, genetic components and genotyping batch	finn-b-I9_ANGINA
Myocardial infarction	FinnGen	NA	2021	11622/187840	Age, sex, genetic components and genotyping batch	finn-b-I9_MI_STRICT
Ischemic stroke	Malik R	29531354	2018	34217/406111	Age and sex	ebi-a-GCST005843
Large-artery atherosclerotic stroke(LAS)	Malik R	29531354	2018	4373/406111	Age and sex	ebi-a-GCST005840
Cardioembolic stroke (CES)	Malik R	29531354	2018	7193/406111	Age and sex	ebi-a-GCST005842

### Screening of instrumental variables (IVs)

The design protocol of this study followed the STROBE-MR guidelines [27]. Using R as the analysis tool, the package “TwoSampleMR V.4.0” was employed for analysis [28, 29]. Setting the parameter r^2^ with a threshold of 0.01 and kilobase pairs (kb) of 5,000 was used to exclude linkage disequilibrium. *P* < 5E-8 was set to screen for significant SNPs. Subsequently, missing SNPs in the resultant database were excluded, and the final IVs obtained were the valid SNPs substantially linked to the exposure factors. The IVs that were weakly associated with the exposure factors may lead to weak instrumental bias. Therefore, the strength of IVs was assessed by the introduction of variance (R^2^) and F-statistics using the following formulas [30]:


$$\begin{array}{l} R^{2}=\frac{2\times MAF\times (1-MAF)\times \beta^{2}}{SE^{2}\times N}\\ F-statistic=\frac{R^{2}\times (N-k-1)}{k^{2}(1-R^{2})} \end{array}$$

In this equation, R^2^ is the cumulative explained variance of the selected SNPs during exposure, MAF is the minor allele frequency, β is the effect value, SE is the standard error, K is the number of SNPs used for the final analysis, and N is the sample size [31]. F > 10 suggests a sufficiently strong relationship between IV and exposure such that the outcomes of MR analysis are protected from weak instrumental bias [30]. Finally, data from the outcome database were extracted for collation and merging, followed by effect allele alignment such that the effect values for exposure and outcome correspond to the same effect allele.

### Statistical analysis

Statistical analyses were carried out on the R program. Mendelian randomization analyses were conducted utilizing the TwoSampleMR package. In this study, there are five methods used to analyze Mendelian randomization results: IVW, Weighted median, MR-Egger, Simple mode, and Weighted mode. IVW meta-analysis, a primary method of analysis, used a random effects model to translate the Wald ratio for each SNP into the effect of each risk factor on the outcome, where the impact of each SNP was shown at a standardized log-transformed exposure level. The weighted median method calculates the causal estimate as a median estimate of the ratio of each genetic variation, weighted by the reciprocal of its variance. The weighted mode assigns causal estimates for each genetic variation by the reciprocal of its variance. Simple mode estimates the causal effect considering each genetic variant individually. Thus, the transformation was done as a weighted regression of SNP outcome effect values on SNP exposure effect values. Subsequently, causal effect estimates (equivalent to beta coefficients) were calculated and converted to odds ratios (ORs). The method provides the highest statistical efficacy provided that the three key assumptions of MR (described in the study design) are met. Because IVW may be subjected to bias or multiple-effect due to the impact of invalid IVs, the validity and robustness of the outcomes were tested by a series of sensitivity analyses.

For sensitivity analysis, MR-Egger regression was first used. This method assumes that the magnitude of the direct effect of the genetic mutation on the outcome (not acting through exposure) is not affected by the impact of the variant on exposure, allowing for an additional intercept (alpha) term that provides an estimate of directional horizontal pleiotropy. To ensure a more reliable analysis of the presence of horizontal pleiotropy, four meta-analyses, including weighted median, simple model, penalized weighted median, and weighted model, were used [32]. In addition, the MR-Egger intercept term was analyzed, and the global Q statistic was calculated to monitor heterogeneity and horizontal pleiotropy further. Finally, a leave-one-out analysis was performed for each SNP to identify IVs that may disproportionately affect the findings of MR analysis.

## RESULTS

### Instrumental variables (IVs)

There were enough AD-associated genome-wide loci (≥2) for MR analysis (number of IVs = 32, R^2^ = 0.057, range of variation in F-statistic 29.82–962.33, total number of cases studied = 63,926) (Supplementary Table 1). In addition, there were sufficient CVD-associated genome-wide loci for reverse MR analysis (IVs: AF = 121, R^2^ = 3.359%, the magnitude of change in F-statistic 30.03–1081.01, total number of studies = 588190 cases; IVs: HF = 12, R^2^ = 0.56%, magnitude of change in F-statistic 30.04–83.1, the total number of cases studied = 977323; IVs: CHD = 16, R^2^ = 0.93%, range of change in F-statistic 30.5–138.9, total number of cases studied = 86995; IVs: AP = 24, R^2^ = 0.599%, range of change in F-statistic 29.64–216.45, the total number of cases studied = 206008 cases; IVs: MI = 15, R^2^ = 0.439%, range of change in F-statistic 30.95–205.15, the total number of cases studied = 199462; IVs: IS = 18, R^2^ = 0.159%, range of change in F-statistic 29.57–66.69, the total number of cases studied = 440328; IVs: LAS = 4, R^2^ = 0.113%, range of change in F-statistic 31.12–61.80, the total number of cases studied = 410484; IVs: CES = 4, R^2^ = 0.166%, range of change in F-statistic 31.69–210.17, the total number of cases studied = 413304) (Supplementary Table 2). The F-statistics were all >10 in the analyses of this study, indicating strong IVs, and no evidence of weak IV bias was found. Thus, these IVs are proven appropriate estimates of the normal impact of exposure and outcome.

### AD affects AP outcomes, whereas AP does not affect AD outcomes

**Figure 2:**
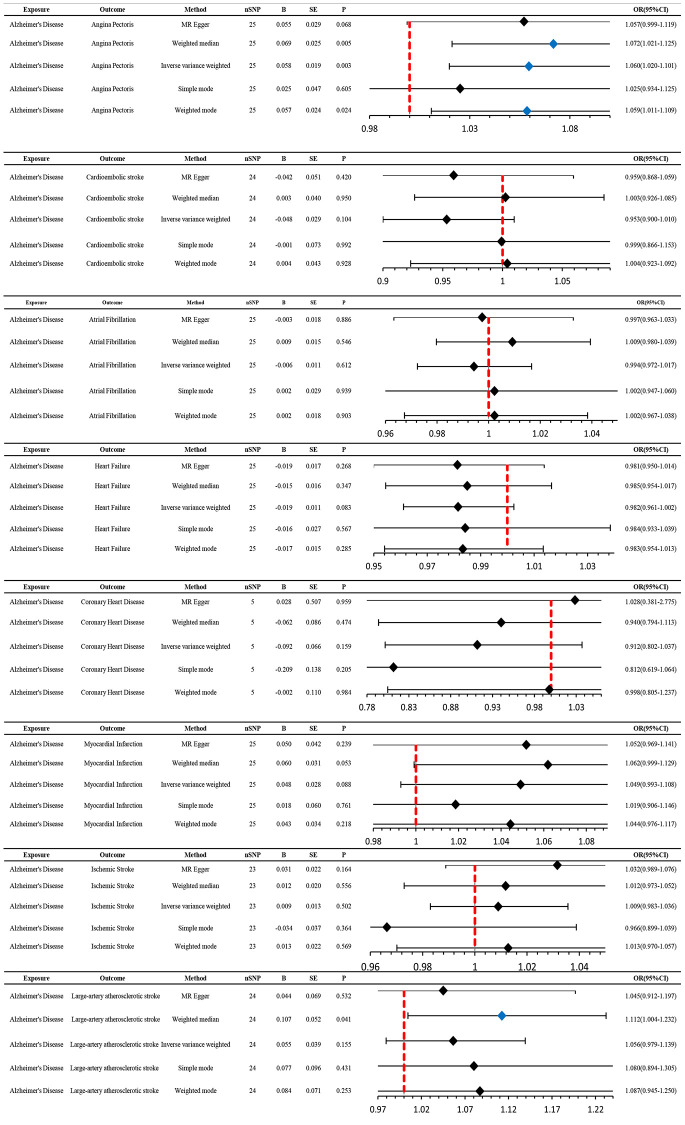
**MR estimates the impact of AD on CVD.** IVW was used as the main method to analyze the two-way causal relationship between AD and CVD. Forest map: Visualize the causal effect of exposure on outcome risk by MR method (when the outcome is cardiovascular disease, i.e., the dichotomy variable, the standard line is the “X = 1” line (orange dashed line)), and the blue markers represent positive results with *P* < 0.05. Abbreviations: AD: Alzheimer’s disease; CVD: cardiovascular disease; IVW: Inverse variance weighting; Beta: risk index; Se: standard error; OR (95% CI): odds ratio (95% confidence interval).

**Figure 3:**
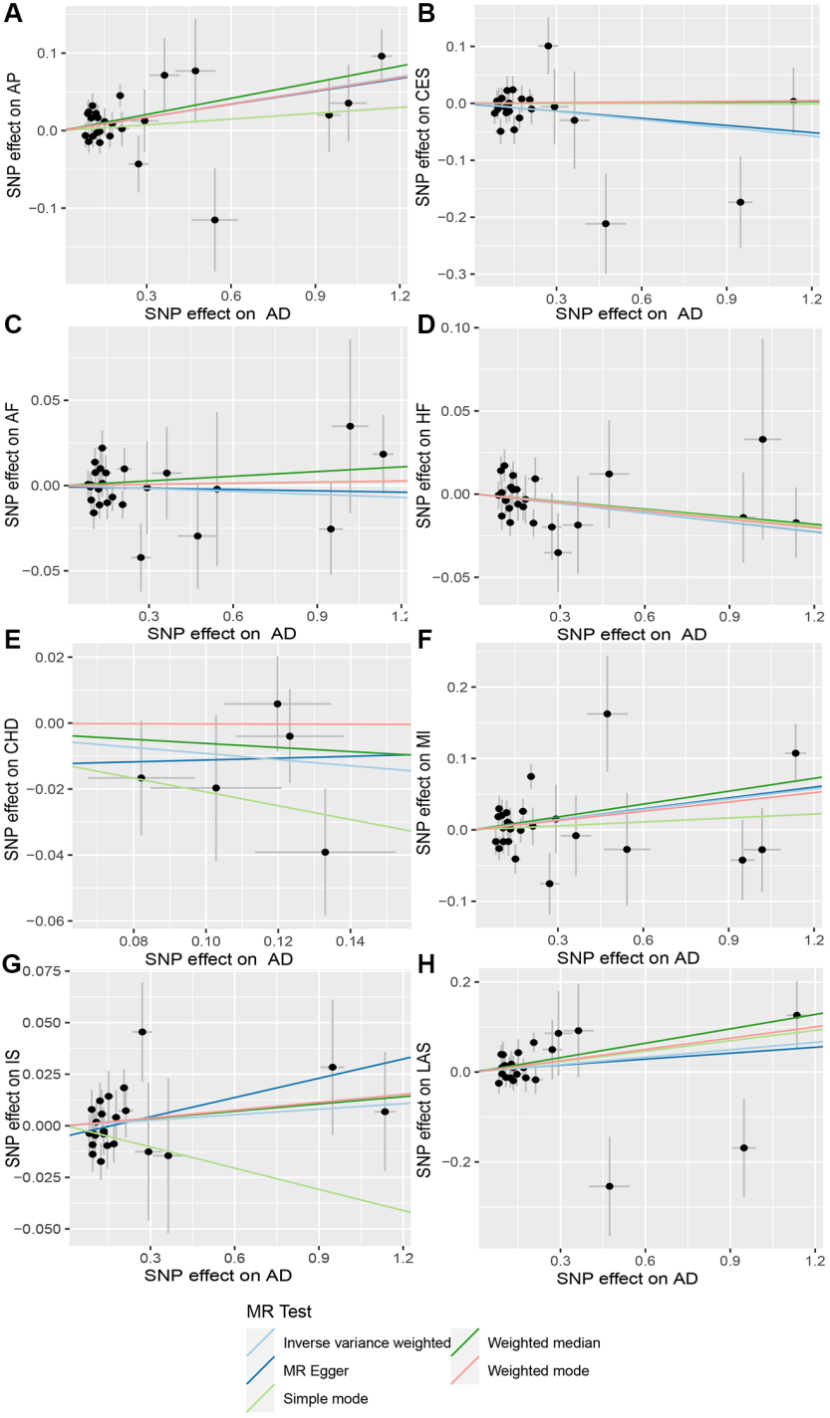
**Scatter plot of AD and CVD.** The horizontal coordinate represents the effect of SNP on exposure when AD is an exposure; the ordinate represents the effect of SNPS on outcomes when CVD is the outcome. (**A**) Exposure: AD, outcome: Angina pectoris (AP); (**B**) Exposure: AD, outcome: Cardioembolic stroke (CES); (**C**) Exposure: AD, outcome: Atrial fibrillation (AF); (**D**) Exposure: AD, outcome: Heart failure (HF); (**E**) Exposure: AD, outcome: Coronary heart disease (CHD); (**F**) Exposure: AD, outcome: Myocardial infarction (MI); (**G**) Exposure: AD, outcome: Ischemic stroke (IS); (**H**) Exposure: AD, outcome: Large-artery atherosclerotic stroke (LAS). Abbreviations: AD: Alzheimer’s disease; CVD: cardiovascular disease.

**Figure 4:**
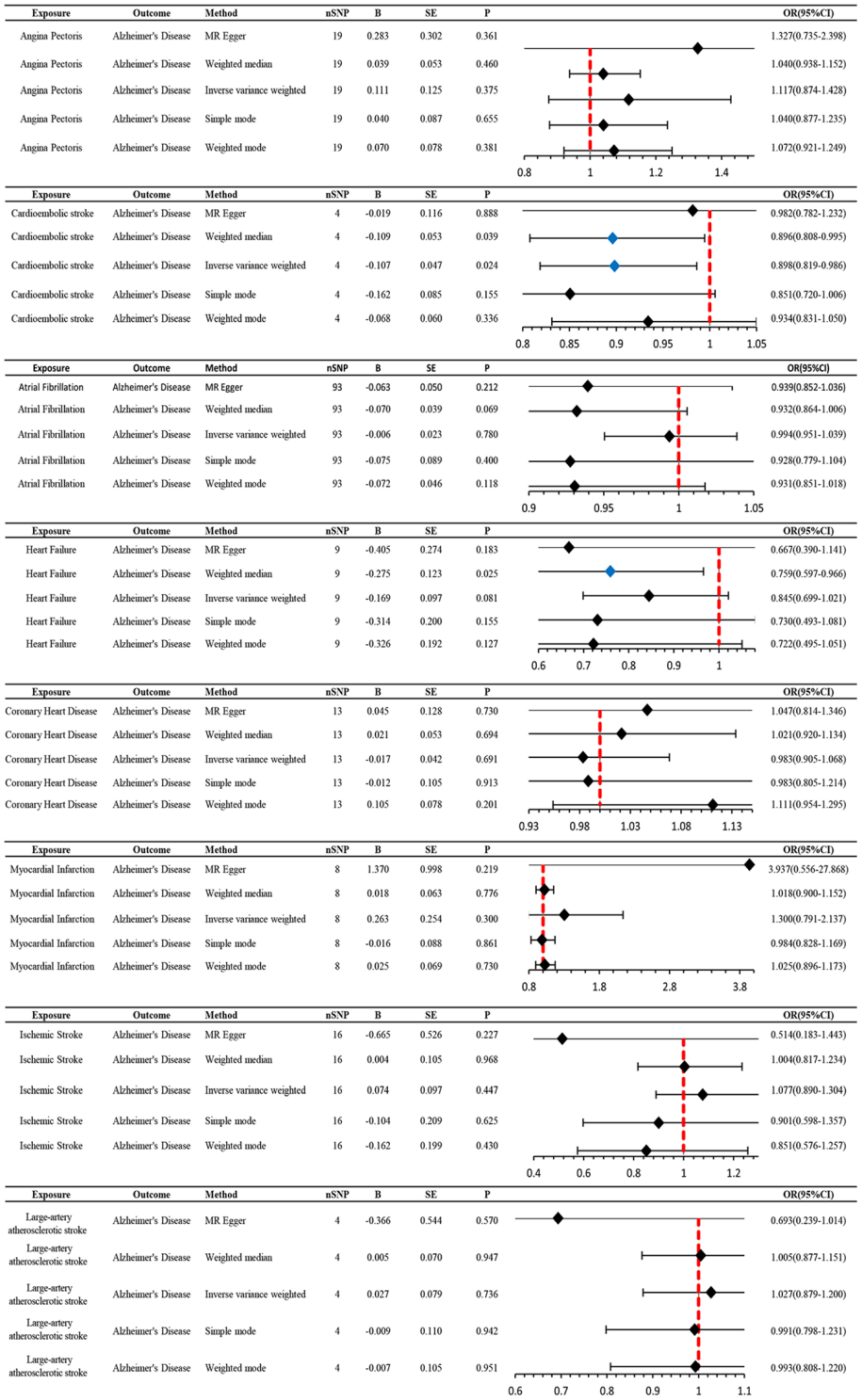
**MR estimates the impact of CVD on AD.** IVW was used as the main method to analyze the two-way causal relationship between CVD and AD. Forest map: Visualize the causal effect of exposure on outcome risk by IVW method (when the outcome is AD, i.e., the dichotomy variable, the standard line is the “X = 1” line (orange dashed line)), and the blue markers represent positive results with *P* < 0.05. Abbreviations: CVD: cardiovascular disease; AD: Alzheimer’s disease; IVW: Inverse variance weighting; Beta: risk index; Se: standard error; OR (95% CI): odds ratio (95% confidence interval).

**Figure 5:**
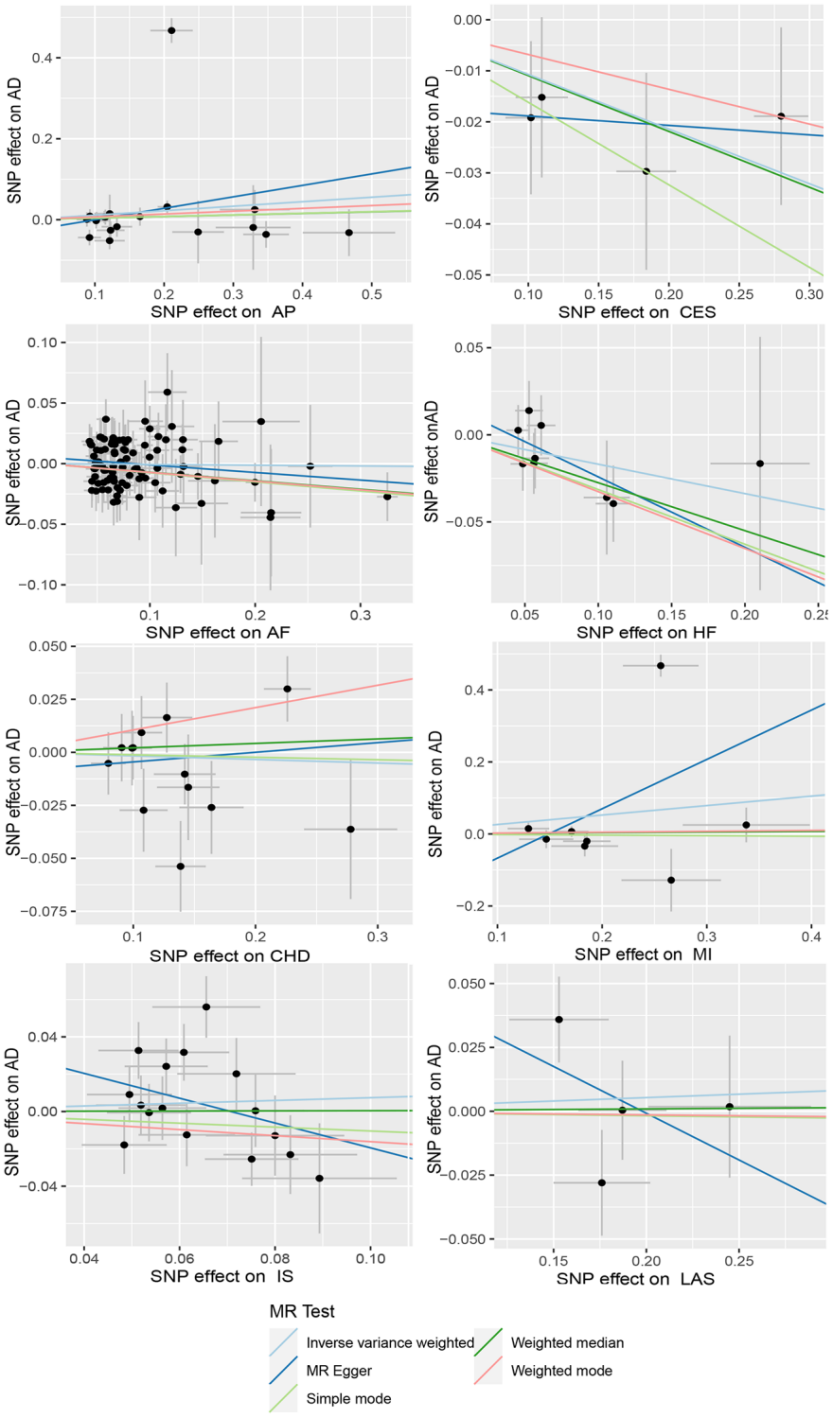
**Scatter plots of CVD and AD.** The horizontal coordinate represents the effect of SNP on exposure when CVD is an exposure; the ordinate represents the effect of SNPS on outcomes when AD is the outcome. Abbreviations: AD: Alzheimer’s disease; CVD: cardiovascular disease; AP: Angina pectoris; CES: Cardioembolic stroke; AF: Atrial fibrillation; HF: Heart failure; CHD: Coronary heart disease; MI: Myocardial infarction; IS: Ischemic stroke; LAS: Large-artery atherosclerotic stroke.

Genetically predicted AD occurrence was significantly associated with a higher incidence of AP (1.06 (1.02–1.10), *P* = 0.003). Likewise, results from both the weighted median (1.07 (1.02–1.12), *P* = 0.005) and weighted model (1.06 (1.01–1.11), *P* = 0.023) demonstrated a significant relationship between the two (Figures 2 and 3). The Cochran *Q* test indicated a lack of heterogeneity among the SNPs for both the IVW method (*P* = 0.15) and MR-Egger method (*P* = 0.12). Similarly, the MR-Egger intercept showed a low likelihood of horizontal pleiotropy (*P* = 0.91) (Table 2). The leave-one-out sensitivity analysis revealed no apparent outliers among the SNPs (Supplementary Figures 1, 2). Conversely, in the reverse MR analysis, there was no genetic correlation between AP and AD (0.99 (0.95–1.04), *P* = 0.78), suggesting that AP does not lead to an increased incidence of AD (Figures 4 and 5, Supplementary Figure 3).

**Table 2:** Heterogeneity and pleiotropy of Alzheimer’s diseases and cardiovascular disease.

**Exposure**	**Outcome**	**Q-statistics**		**Pleiotropic test**	
		**MR egger**	**IVW**	**egger_intercept**	***p* val**
Alzheimer’s diseases	Angina pectoris	Q = 31.2744487923026 P = 0.116198516999501	Q = 31.2913710103507 P = 0.1455579158033620	7.01E-04	9.12E-01
Alzheimer’s diseases	Ischemic stroke (cardioembolic stroke)	Q = 27.3018384090908 P = 0.19998596940784	Q = 27.329536923561 P = 0.242200605399031	−1.32E-03	8.83E-01
Alzheimer’s diseases	Atrial fibrillation	Q = 27.1390177529223 P = 0.250097119842232	Q = 27.2048400213745 P = 0.29499585079596	−8.06E-04	8.15E-01
Alzheimer’s diseases	Heart failure	Q = 24.497030623955 P = 0.376728459266513	Q = 24.4975016553616 P = 0.433462882001429	6.88E-05	9.83E-01
Alzheimer’s diseases	Coronary heart disease	Q = 3.98253152246554 P = 0.263356598621039	Q = 4.05912810236342 P = 0.39806287073209	−1.40E-02	8.26E-01
Alzheimer’s diseases	Myocardial infarction	Q = 46.3338873124734 P = 0.00271774043738455	Q = 46.3482050834492 P = 0.00401939321275194	−7.62E-04	9.34E-01
Alzheimer’s diseases	Ischemic stroke	Q = 21.144962355489 P = 0.450122266434693	Q = 22.8293327453125 P = 0.411419707241582	−4.96E-03	2.10E-01
Alzheimer’s diseases	Ischemic stroke (large-artery atherosclerotic stroke)	Q = 30.9989316046781 P = 0.0961373949725882	Q = 31.0502693988936 P = 0.121563116697403	2.22E-03	8.50E-01
Angina pectoris	Alzheimer’s diseases	Q = 238.934342189307 P = 3.768252909456E-41	Q = 244.50194182614 P = 1.06833415049394E-41	−2.84E-02	5.37E-01
Ischemic stroke (cardioembolic stroke)	Alzheimer’s diseases	Q = 0.327418217777312 P = 0.848988949302951	Q = 1.02700746484242 P = 0.794717332987937	−1.70E-02	4.91E-01
Atrial fibrillation	Alzheimer’s diseases	Q = 73.0064793610771 P = 0.91669069589157	Q = 74.6143811377799 P = 0.907031206270659	5.25E-03	2.08E-01
Heart failure	Alzheimer’s diseases	Q = 4.07814530131693 P = 0.770733899232561	Q = 4.92389931945689 P = 0.765678073985451	1.63E-02	3.88E-01
Coronary heart disease	Alzheimer’s diseases	Q = 16.4524193832707 P = 0.125148555159931	Q = 16.8513813779198 P = 0.155273889596569	−9.07E-03	6.16E-01
Myocardial infarction	Alzheimer’s diseases	Q = 171.264349935542 P = 2.42552212550759E-34	Q = 208.710647570382 P = 1.63756035918218E-41	−2.04E-01	2.96E-01
Ischemic stroke	Alzheimer’s diseases	Q = 27.5468742587226 P = 0.0163307254683385	Q = 31.5563413545666 P = 0.00739484569866784	4.70E-02	1.75E-01
Ischemic stroke (large-artery atherosclerotic stroke)	Alzheimer’s diseases	Q = 4.86550958684538 P = 0.0877946430392627	Q = 6.16740101104067 P = 0.103743844741416	7.24E-02	5.41E-01

### CES is a protective factor for AD, whereas AD does not affect CES

We utilized MR analysis to investigate the causal relationship between CES and AD. The results demonstrated a significant causal relationship between CES and AD (0.9 (0.82–0.99), *P* = 0.02) (Figures 4 and 5). The weighted median analysis also indicated that the incidence of CES led to a decrease in the occurrence of AD (0.9 (0.851–0.99)) (Figure 4). The *P*-values for the IVW-Q test and the MR Egger-Q test were 0.7 and 0.8, respectively, suggesting a lack of horizontal pleiotropy among the single nucleotide polymorphisms (SNPs), thus providing robust results (Table 2). However, no causal relationship was found between AD and CES (0.95 (0.9–1.0), *P* = 0.1), indicating non-significant results (Figure 2).

### No causal relationship is observed between AD and other CVDs

When AD was the exposure factor and another CVD was the outcome, the IVW method calculated the following results: AF: OR = 0.99 (95% CI 0.97–1.02), *P* = 0.61; HF: OR = 0.98 (95% CI 0.96–1.00), *P* = 0.08; CHD: OR = 0.91 (95% CI 0.80–1.04), *P* = 0.16; MI: OR = 1.05 (95% CI 0.99–1.11), *P* = 0.09; IS: OR = 1.01 (95% CI 0.98–1.04), *P* = 0.50; LAS: OR = 1.06 (95% CI 0.98–1.04) None of these results showed significant differences. Forest plots of Mendelian randomization effects for individual SNPs were plotted (Supplementary Figure 4). The results obtained from the inverse MR analysis were also not statistically significant (AF → AD: (OR (95% CI): 0.99 (0.95–1.04), *P* = 0.78); HF → AD: (OR (95% CI): 0.84 (0.70–1.02), *P* = 0.08); CHD → AD: (OR (95% CI): 0.98 (0.91–1.07), *P* = 0.69); MI → AD: (OR (95% CI): 1.30 (0.79–2.14), *P* = 0.30); IS → AD: (OR(95% CI): 1.08 (0.89–1.30), *P* = 0.45); LAS → AD: (OR(95% CI):1.03 (95% CI 0.88–1.20), *P* = 0.74)). The results of other analytical methods are shown in (Figures 2 and 4). In addition, a scatter plot shows genetic visualization estimates of AD for CVD (Figures 3 and 5).

## DISCUSSION

### Principal findings

In this study, we leveraged large consortium and genome-wide association study (GWAS) summary data to explore the bidirectional causality between Alzheimer’s disease (AD) and cardiovascular disease (CVD). We unveiled a significant association between the two. When AD was considered as exposure and angina pectoris (AP) as outcome, the effect estimate ranged from 1.02 to 1.10, suggesting a causal relationship between genetic predisposition to AD and increased risk of AP. While the relative risk increase might not seem substantial, it still holds significant epidemiological and clinical implications. Furthermore, when cardioembolic stroke (CES) was the exposure and AD the outcome, the effect estimate ranged from 0.82 to 0.99. This result suggests a causal relationship between a genetic predisposition to CES and a reduced risk of AD, hinting at a possible protective role of CES in lowering the incidence of AD.

### Comparison with other studies

Our findings are consistent with a previous Mendelian randomization study, which found no causal relationship between atrial fibrillation and the risk of AD [13]. However, in stark contrast to our conclusion, a large-scale multi-ancestry stroke GWAS meta-analysis of stroke data and International Genomics of Alzheimer’s Project data found no causal relationship between genetically correlated CES and AD [33]. The discrepancy might be attributed to potential confounders, the selection of study populations, and disease data selection bias.

### Possible mechanisms

AD may increase the risk of CVD, particularly AP, possibly due to the action of beta-amyloid (Aβ) peptides. As one of the pathological hallmarks of AD, amyloid deposition in the heart triggers inflammatory reactions and organ dysfunction [34]. The APOE4 gene, reportedly associated with AD [35], exerts significant impacts on lipid metabolism, thereby serving as a risk factor for CVD. The APOE locus is a common influence on the genetic structure of coronary artery disease and AD, and the association between the two vanishes when the impact of the APOE gene is excluded [36].

The mechanism by which genetically predicted cardioembolic stroke reduces the risk of AD may involve risk factor control, pharmacological interventions, and lifestyle changes. The treatment and management of cardioembolic stroke often involve improving cardiovascular health, which may enhance cerebral blood flow and reduce microvascular occlusions, thereby preventing the development of AD [37, 38]. Therapeutic drugs, such as aspirin and other antiplatelet drugs [39, 40], and rivaroxaban and other anticoagulants [41], might ameliorate cerebral microcirculation, alleviate neuroinflammation, and counteract Aβ deposition, thus decelerating the progression of AD. A diagnosis of cardioembolic stroke may prompt patients to make lifestyle changes, such as increasing physical activity, improving dietary habits, and reducing smoking, which might enhance brain health and reduce the incidence of AD [42].

### Strengths and limitations

This study utilized large GWAS datasets from AD and CVD, allowing for a systematic investigation of the causal relationship between genetically predisposed AD and CVD, while avoiding the confounding effects of reverse causality and potential covariates. The variety of analytical methods used in this study enhances the accuracy and reproducibility of the results. Furthermore, a series of additional sensitivity analyses ensured the robustness of the findings.

Despite these strengths, certain limitations should be acknowledged. First, the selected samples were from European cohorts, excluding other populations such as Asian and American populations. This limitation may reduce the generalizability of the findings to other populations. Second, the limitations associated with database statistics are evident. Limited statistical data, coupled with the lack of comprehensive raw data information (e.g., inclusion/exclusion criteria, interventions), could adversely impact the accuracy of the results. Third, as Mendelian randomization studies continue to evolve, the use of additional statistical methods such as multivariable and mediation analyses can assist in mitigating the interference from factors such as medication, smoking, and lifestyle, thereby enhancing the accuracy of the research results on the causal relationship between AD and CVD. Fourth, the effect estimates in this study are relatively low, which could be related to the sample size of the disease group. As research into these diseases continues to advance, incorporating more GWAS data from diverse populations could help address this issue.

## CONCLUSION

Conclusively, this study suggests that AD increases the risk of AP and that CES is a protective factor for AD. However, no conclusive evidence suggested a causal association between other CVDs and AD. Therefore, more rigorous clinical and laboratory studies are needed for further investigation.

## Supplementary Materials

Supplementary Figures

Supplementary Table 1

Supplementary Table 2
