# Supplementary material for: Causal relationship between Alzheimer’s disease and cardiovascular disease: a bidirectional Mendelian randomization analysis
Source: Aging (Albany NY). 2023 Sep 2;15(17):9022–40. doi: 10.18632/aging.205013 (PMC10522384; doi:10.18632/aging.205013)
Supplement: Supplementary Figures [file aging-15-205013-s001.pdf]

SUPPLEMENTARY FIGURES

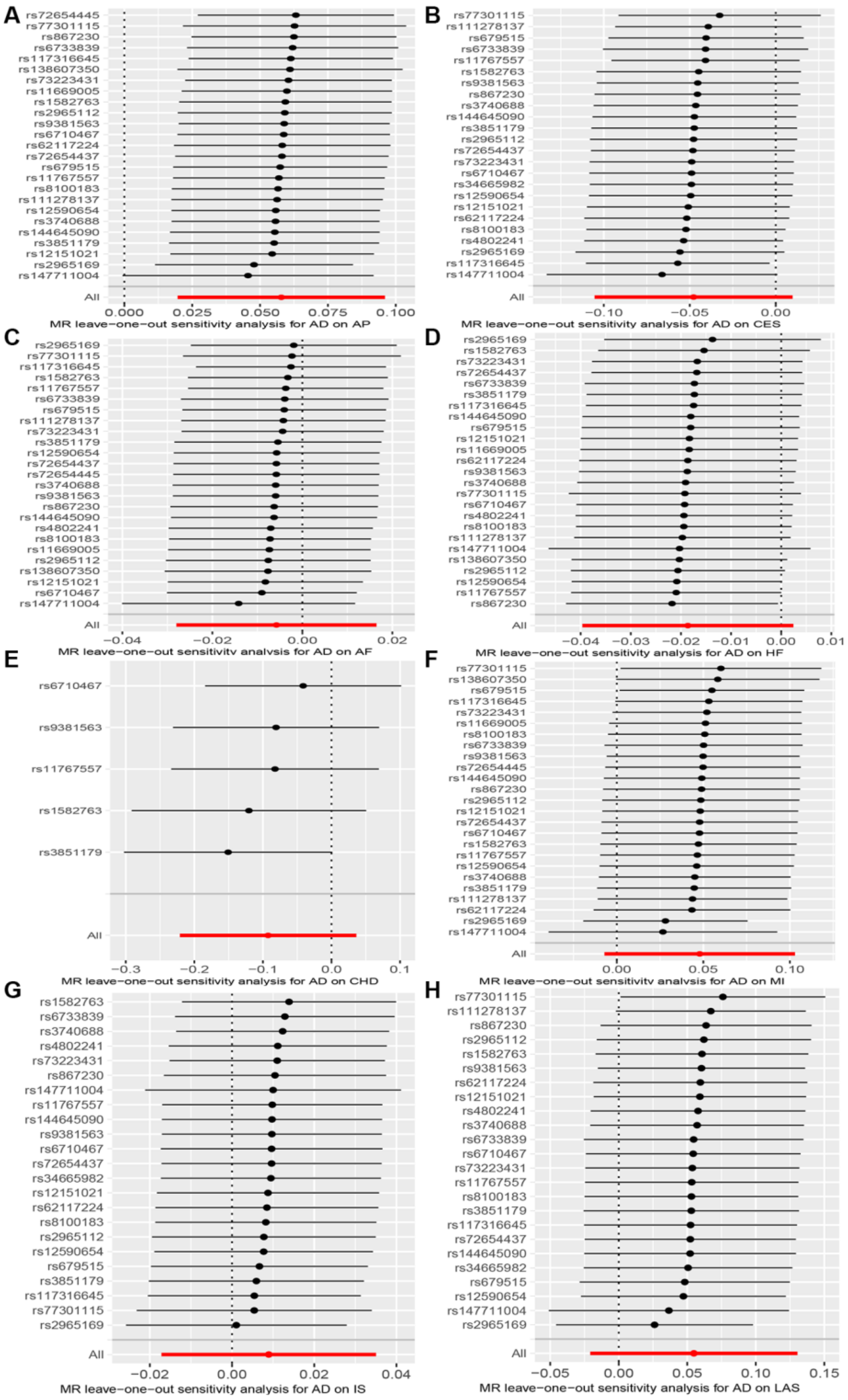

Supplementary Figure 1. MR leave-one-out analysis plots for the relationships of AD with CVD. (**A**) Exposure: AD, outcome: Angina pectoris (AP); (**B**) Exposure: AD, outcome: Cardioembolic stroke (CES); (**C**) Exposure: AD, outcome: Atrial fibrillation (AF); (**D**) Exposure: AD, outcome: Heart failure (HF); (**E**) Exposure: AD, outcome: Coronary heart disease (CHD); (**F**) Exposure: AD, outcome: Myocardial infarction (MI); (**G**) Exposure: AD, outcome: Ischemic stroke (IS); (**H**) Exposure: AD, outcome: Large-artery atherosclerotic stroke (LAS). Abbreviations: AD: Alzheimer's disease; CVD: cardiovascular disease.

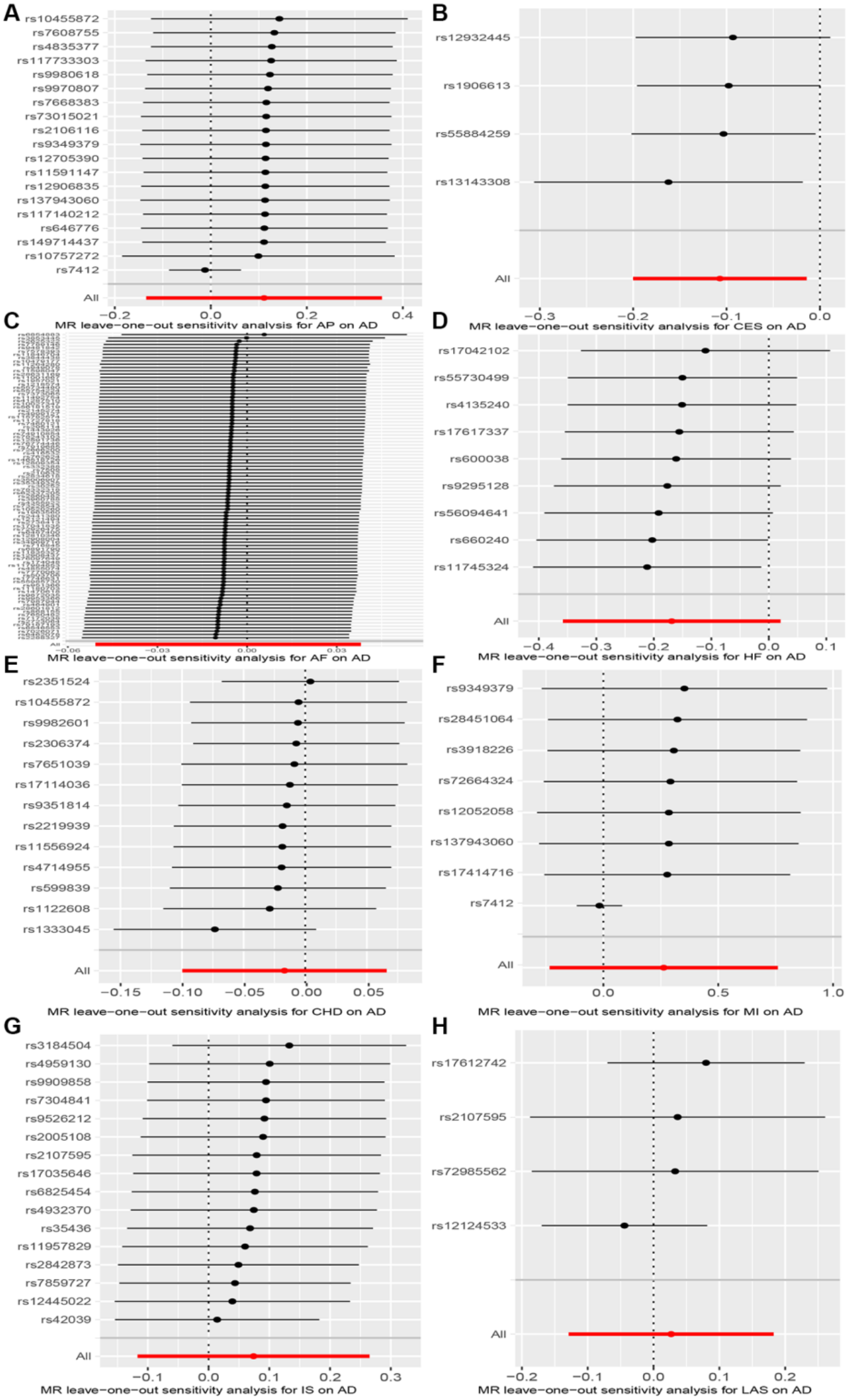

Supplementary Figure 2. MR leave-one-out analysis plots for the relationships of CVD with AD. (**A**) Exposure: Angina pectoris (AP), outcome: Alzheimer's disease (AD); (**B**) Exposure: Cardioembolic stroke (CES), outcome: Alzheimer's disease (AD); (**C**) Exposure: Atrial fibrillation (AF), outcome: Alzheimer's disease (AD); (**D**) Exposure: AD, outcome: Heart failure (HF); (**E**) Exposure: Coronary heart disease (CHD), outcome: Alzheimer's disease (AD); (**F**) Exposure: Myocardial infarction (MI), outcome: Alzheimer's disease (AD); (**G**) Exposure: Ischemic stroke (IS), outcome: Alzheimer's disease (AD); (**H**) Exposure: Large-artery atherosclerotic stroke (LAS), outcome: Alzheimer's disease (AD). Abbreviation: CVD; cardiovascular disease.

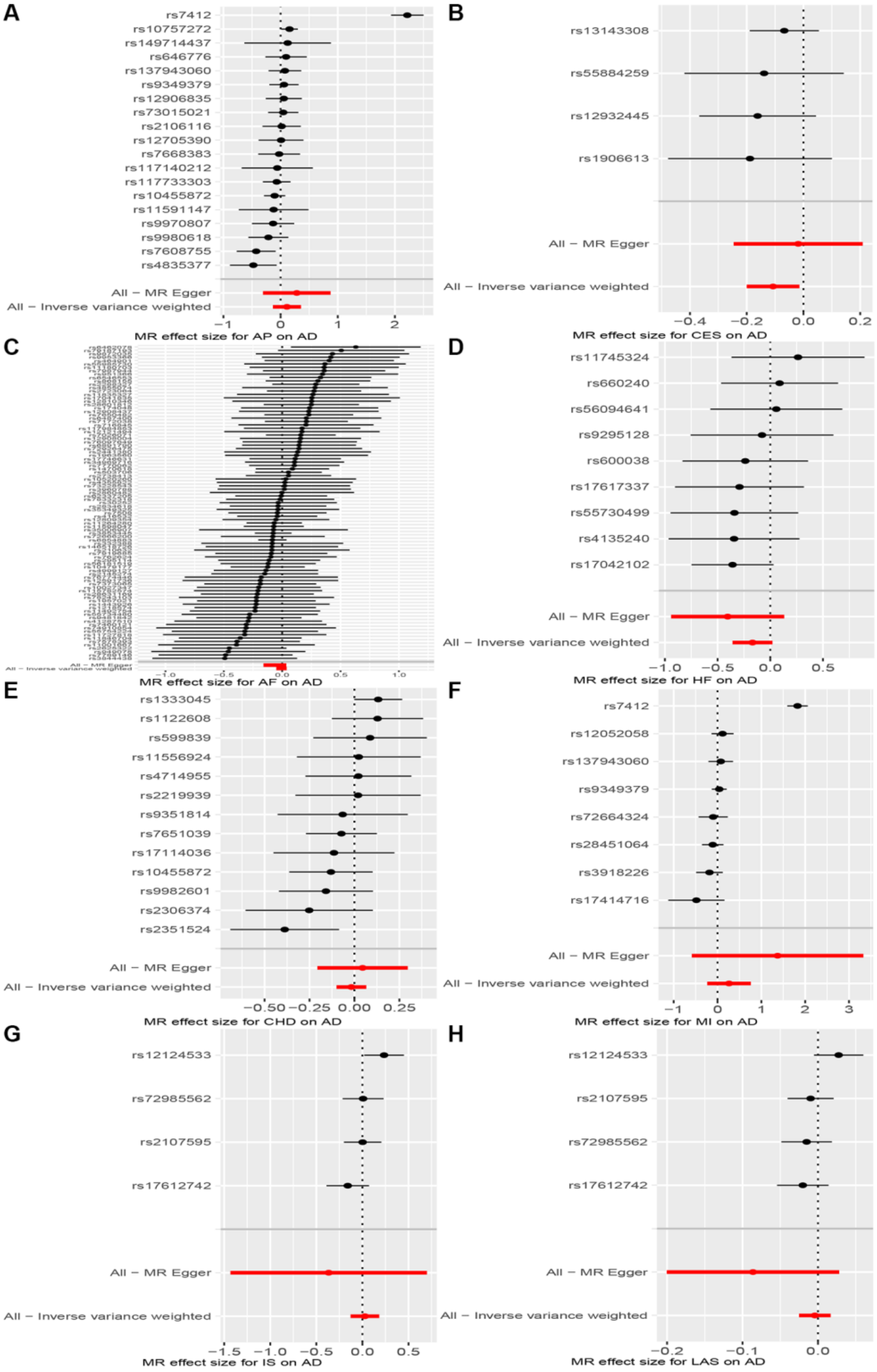

Supplementary Figure 3. Mendelian randomization effects forest plots for individual SNPs with CVD as the exposure, AD as the outcome. This figure showed the forest plots for individual SNPs with CVD as the exposure, AD as the outcome. The dark red marker points indicated All-IVW and All-MR Egger. (**A**) Exposure: Angina pectoris (AP), outcome: Alzheimer's disease (AD); (**B**) Exposure: Cardioembolic stroke (CES), outcome: Alzheimer's disease (AD); (**C**) Exposure: Atrial fibrillation (AF), outcome: Alzheimer's disease (AD); (**D**) Exposure: AD, outcome: Heart failure (HF); (**E**) Exposure: Coronary heart disease (CHD), outcome: Alzheimer's disease (AD); (**F**) Exposure: Myocardial infarction (MI), outcome: Alzheimer's disease (AD); (**G**) Exposure: Ischemic stroke (IS), outcome: Alzheimer's disease (AD); (**H**) Exposure: Large-artery atherosclerotic stroke (LAS), outcome: Alzheimer's disease (AD). Abbreviation: CVD: cardiovascular disease.

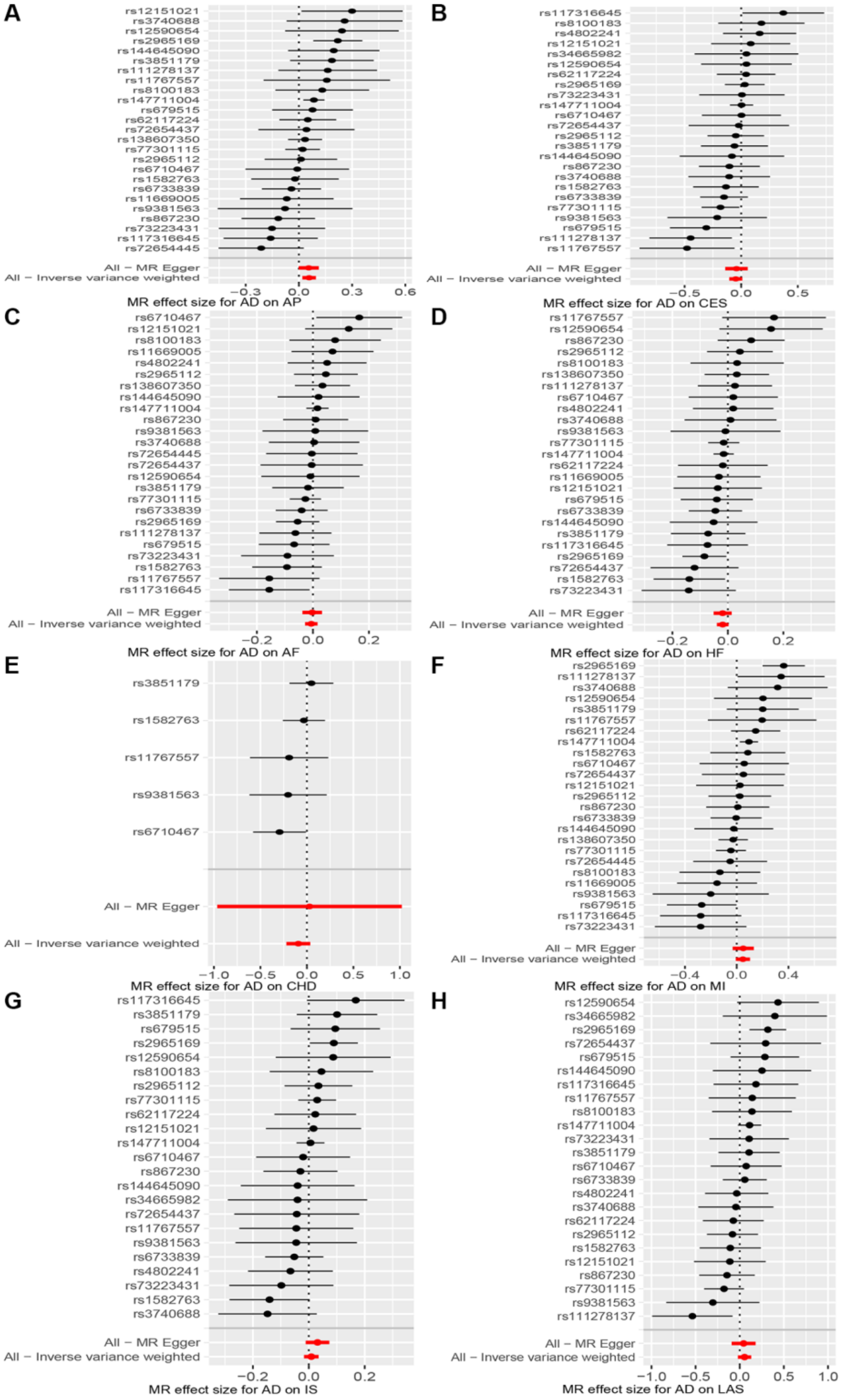

Supplementary Figure 4. Mendelian randomization effects forest plots for individual SNPs with AD as the exposure, CVD as the outcome. This figure showed the forest plots for individual SNPs with AD as the exposure, CVD as the outcome. The dark red marker points indicated All-IVW and All-MR Egger. (**A**) Exposure: AD, outcome: Angina pectoris (AP); (**B**) Exposure: AD, outcome: Cardioembolic stroke (CES); (**C**) Exposure: AD, outcome: Atrial fibrillation (AF); (**D**) Exposure: AD, outcome: Heart failure (HF); (**E**) Exposure: AD, outcome: Coronary heart disease (CHD); (**F**) Exposure: AD, outcome: Myocardial infarction (MI); (**G**) Exposure: AD, outcome: Ischemic stroke (IS); (**H**) Exposure: AD, outcome: Large-artery atherosclerotic stroke (LAS). Abbreviations: AD: Alzheimer's disease; CVD: cardiovascular disease.
