# Supplementary material for: Causal relationship between Alzheimer’s disease and cardiovascular disease: a bidirectional Mendelian randomization analysis
Source: Aging (Albany NY). 2023 Sep 2;15(17):9022–40. doi: 10.18632/aging.205013 (PMC10522384; doi:10.18632/aging.205013)
Supplement: Supplementary Table 1 [file aging-15-205013-s002.docx]

**Supplementary Table 1. SNPs information of Alzheimer's disease with cardiovascular disease.**

|  |  |  | **Alzheimer's Disease** | | | | |
| --- | --- | --- | --- | --- | --- | --- | --- |
| **SNP** | **effect allele** | **other allele** | **Beta.exposure** | **se.exposure** | **pval.exposure** | **R2** | **F** |
| rs679515 | C | T | -0.1508 | 0.0183 | 1.55E-16 | 1.06E-03 | 6.79E+01 |
| rs6710467 | A | G | 0.133 | 0.0195 | 8.96E-12 | 7.30E-04 | 4.65E+01 |
| rs6733839 | T | C | 0.1693 | 0.0154 | 4.02E-28 | 1.89E-03 | 1.21E+02 |
| rs9381563 | T | C | -0.0821 | 0.0148 | 2.93E-08 | 4.80E-04 | 3.08E+01 |
| rs114812713 | C | G | 0.298 | 0.0431 | 4.47E-12 | 7.50E-04 | 4.78E+01 |
| rs34665982 | C | T | -0.0967 | 0.0166 | 5.80E-09 | 5.30E-04 | 3.39E+01 |
| rs11767557 | C | T | -0.1028 | 0.0182 | 1.56E-08 | 5.00E-04 | 3.19E+01 |
| rs73223431 | T | C | 0.0936 | 0.0153 | 8.34E-10 | 5.90E-04 | 3.74E+01 |
| rs867230 | A | C | 0.1333 | 0.0158 | 3.49E-17 | 1.11E-03 | 7.12E+01 |
| rs11257242 | G | C | 0.0841 | 0.0154 | 4.64E-08 | 4.70E-04 | 2.98E+01 |
| rs3740688 | T | G | 0.0935 | 0.0144 | 9.70E-11 | 6.60E-04 | 4.22E+01 |
| rs3851179 | C | T | 0.1198 | 0.0148 | 5.81E-16 | 1.02E-03 | 6.55E+01 |
| rs1582763 | A | G | -0.1232 | 0.0149 | 1.19E-16 | 1.07E-03 | 6.84E+01 |
| rs12590654 | A | G | -0.0906 | 0.0157 | 8.73E-09 | 5.20E-04 | 3.33E+01 |
| rs12151021 | G | A | -0.1071 | 0.0169 | 2.56E-10 | 6.30E-04 | 4.02E+01 |
| rs77301115 | A | G | 0.9486 | 0.0427 | 2.25E-109 | 7.66E-03 | 4.94E+02 |
| rs117316645 | A | G | 0.2709 | 0.0349 | 8.95E-15 | 9.40E-04 | 6.03E+01 |
| rs75463276 | G | C | 0.4064 | 0.0618 | 4.67E-11 | 6.80E-04 | 4.32E+01 |
| rs2965112 | G | A | -0.212 | 0.0261 | 4.40E-16 | 1.03E-03 | 6.60E+01 |
| rs72654445 | A | G | -0.5425 | 0.0811 | 2.27E-11 | 7.00E-04 | 4.47E+01 |
| rs62117224 | A | G | -0.1773 | 0.0188 | 3.66E-21 | 1.39E-03 | 8.89E+01 |
| rs11669005 | A | G | 0.1085 | 0.0164 | 4.15E-11 | 6.80E-04 | 4.38E+01 |
| rs2965169 | C | A | -0.2056 | 0.0161 | 2.75E-37 | 2.54E-03 | 1.63E+02 |
| rs138607350 | G | T | 1.0175 | 0.0651 | 4.80E-55 | 3.81E-03 | 2.44E+02 |
| rs11668327 | C | G | -0.4126 | 0.0223 | 2.65E-76 | 5.33E-03 | 3.42E+02 |
| rs8100183 | T | C | 0.1261 | 0.0202 | 4.52E-10 | 6.10E-04 | 3.90E+01 |
| rs144645090 | A | G | 0.3633 | 0.0533 | 9.04E-12 | 7.30E-04 | 4.65E+01 |
| rs147711004 | A | G | 1.1354 | 0.0366 | 1.00E-200 | 1.48E-02 | 9.62E+02 |
| rs4802241 | C | A | -0.146 | 0.0211 | 4.26E-12 | 7.50E-04 | 4.79E+01 |
| rs3112439 | C | G | -0.2677 | 0.0215 | 1.72E-35 | 2.42E-03 | 1.55E+02 |
| rs111278137 | A | G | -0.4735 | 0.0713 | 3.20E-11 | 6.90E-04 | 4.41E+01 |
| rs72654437 | A | G | -0.2929 | 0.049 | 2.28E-09 | 5.60E-04 | 3.57E+01 |

|  |  |  | **Atrial Fibrillation** | | |
| --- | --- | --- | --- | --- | --- |
| **SNP** | **effect allele** | **other allele** | **Beta.outcome** | **se.outcome** | **pval.outcome** |
| rs679515 | C | T | 0.0101 | 0.0097 | 2.99E-01 |
| rs6710467 | A | G | 0.022 | 0.0104 | 3.37E-02 |
| rs6733839 | T | C | -0.0068 | 0.008 | 3.94E-01 |
| rs9381563 | T | C | -0.0007 | 0.0079 | 9.28E-01 |
| rs114812713 | C | G | #N/A | #N/A | #N/A |
| rs34665982 | C | T | #N/A | #N/A | #N/A |
| rs11767557 | C | T | 0.016 | 0.0094 | 8.74E-02 |
| rs73223431 | T | C | -0.0085 | 0.0079 | 2.79E-01 |
| rs867230 | A | C | 0.0013 | 0.0079 | 8.65E-01 |
| rs11257242 | G | C | #N/A | #N/A | #N/A |
| rs3740688 | T | G | 0.0004 | 0.0077 | 9.54E-01 |
| rs3851179 | C | T | -0.0021 | 0.0078 | 7.84E-01 |
| rs1582763 | A | G | 0.0114 | 0.0078 | 1.47E-01 |
| rs12590654 | A | G | 0.0008 | 0.0081 | 9.26E-01 |
| rs12151021 | G | A | -0.0137 | 0.0085 | 1.06E-01 |
| rs77301115 | A | G | -0.0255 | 0.027 | 3.44E-01 |
| rs117316645 | A | G | -0.0422 | 0.0199 | 3.42E-02 |
| rs75463276 | G | C | #N/A | #N/A | #N/A |
| rs2965112 | G | A | -0.0098 | 0.0123 | 4.26E-01 |
| rs72654445 | A | G | 0.0021 | 0.0451 | 9.64E-01 |
| rs62117224 | A | G | #N/A | #N/A | #N/A |
| rs11669005 | A | G | 0.0076 | 0.0081 | 3.46E-01 |
| rs2965169 | C | A | 0.0112 | 0.0081 | 1.63E-01 |
| rs138607350 | G | T | 0.0348 | 0.0511 | 4.96E-01 |
| rs11668327 | C | G | #N/A | #N/A | #N/A |
| rs8100183 | T | C | 0.01 | 0.0105 | 0.3387 |
| rs144645090 | A | G | 0.0073 | 0.0271 | 0.7864 |
| rs147711004 | A | G | 0.0184 | 0.023 | 0.4248 |
| rs4802241 | C | A | -0.0074 | 0.0105 | 0.4762 |
| rs3112439 | C | G | #N/A | #N/A | #N/A |
| rs111278137 | A | G | 0.0296 | 0.0311 | 0.3413 |
| rs72654437 | A | G | 0.0013 | 0.0273 | 0.9626 |

|  |  |  | **Heart Failure** | | |
| --- | --- | --- | --- | --- | --- |
| **SNP** | **effect allele** | **other allele** | **Beta.outcome** | **se.outcome** | **pval.outcome** |
| rs679515 | C | T | 0.006 | 0.01 | 0.5527 |
| rs6710467 | A | G | 0.0026 | 0.0109 | 0.8096 |
| rs6733839 | T | C | -0.0076 | 0.0083 | 0.3603 |
| rs9381563 | T | C | 0.0007 | 0.0083 | 0.9364 |
| rs114812713 | C | G | #N/A | #N/A | #N/A |
| rs34665982 | C | T | #N/A | #N/A | #N/A |
| rs11767557 | C | T | -0.0171 | 0.0098 | 0.08175 |
| rs73223431 | T | C | -0.0132 | 0.0081 | 0.1047 |
| rs867230 | A | C | 0.0112 | 0.0082 | 0.1702 |
| rs11257242 | G | C | #N/A | #N/A | #N/A |
| rs3740688 | T | G | 0.0009 | 0.0079 | 0.9048 |
| rs3851179 | C | T | -0.0085 | 0.0082 | 0.2974 |
| rs1582763 | A | G | 1.71E-02 | 0.0081 | 0.03606 |
| rs12590654 | A | G | -0.0141 | 0.0086 | 0.102 |
| rs12151021 | G | A | 0.0039 | 0.0087 | 0.6559 |
| rs77301115 | A | G | -0.0141 | 0.0271 | 0.6017 |
| rs117316645 | A | G | -0.0198 | 0.0201 | 0.3236 |
| rs75463276 | G | C | #N/A | #N/A | #N/A |
| rs2965112 | G | A | -0.0092 | 0.0128 | 0.4742 |
| rs72654445 | A | G | #N/A | #N/A | #N/A |
| rs62117224 | A | G | 0.0032 | 0.0146 | 0.8255 |
| rs11669005 | A | G | -0.0035 | 0.0083 | 0.6731 |
| rs2965169 | C | A | 0.0174 | 0.0083 | 0.03571 |
| rs138607350 | G | T | 0.0329 | 0.0603 | 0.5857 |
| rs11668327 | C | G | #N/A | #N/A | #N/A |
| rs8100183 | T | C | 0.0042 | 0.0108 | 0.6963 |
| rs144645090 | A | G | -0.0186 | 0.0293 | 0.525 |
| rs147711004 | A | G | -0.0172 | 0.0212 | 0.4181 |
| rs4802241 | C | A | -0.0028 | 0.0108 | 0.7987 |
| rs3112439 | C | G | #N/A | #N/A | #N/A |
| rs111278137 | A | G | -0.0121 | 0.0323 | 0.7086 |
| rs72654437 | A | G | 0.0352 | 0.0237 | 0.138 |

|  |  |  | **Myocardial Infarction** | | |
| --- | --- | --- | --- | --- | --- |
| **SNP** | **effect allele** | **other allele** | **Beta.outcome** | **se.outcome** | **pval.outcome** |
| rs679515 | C | T | 0.0408 | 0.021 | 4.845E-02 |
| rs6710467 | A | G | 0.0077 | 0.023 | 7.426E-01 |
| rs6733839 | T | C | -0.0008 | 0.017 | 9.629E-01 |
| rs9381563 | T | C | 0.0165 | 0.019 | 3.805E-01 |
| rs114812713 | C | G | #N/A | #N/A | #N/A |
| rs34665982 | C | T | #N/A | #N/A | #N/A |
| rs11767557 | C | T | -0.0202 | 0.022 | 3.599E-01 |
| rs73223431 | T | C | -0.0261 | 0.017 | 1.223E-01 |
| rs867230 | A | C | 0.0011 | 0.017 | 9.494E-01 |
| rs11257242 | G | C | #N/A | #N/A | #N/A |
| rs3740688 | T | G | 0.0296 | 0.018 | 1.081E-01 |
| rs3851179 | C | T | 0.0241 | 0.017 | 1.555E-01 |
| rs1582763 | A | G | -0.0106 | 0.018 | 5.583E-01 |
| rs12590654 | A | G | -0.0184 | 0.018 | 2.921E-01 |
| rs12151021 | G | A | -0.0027 | 0.019 | 8.849E-01 |
| rs77301115 | A | G | -0.0424 | 0.056 | 4.494E-01 |
| rs117316645 | A | G | -7.54E-02 | 0.043 | 8.240E-02 |
| rs75463276 | G | C | #N/A | #N/A | #N/A |
| rs2965112 | G | A | -0.0051 | 0.026 | 8.458E-01 |
| rs72654445 | A | G | 0.0274 | 0.079 | 7.285E-01 |
| rs62117224 | A | G | -0.026 | 0.017 | 1.296E-01 |
| rs11669005 | A | G | -0.0165 | 0.017 | 3.364E-01 |
| rs2965169 | C | A | -0.0747 | 0.017 | 1.220E-05 |
| rs138607350 | G | T | -0.0278 | 0.059 | 6.371E-01 |
| rs11668327 | C | G | #N/A | #N/A | #N/A |
| rs8100183 | T | C | -0.0164 | 0.0201 | 0.4148 |
| rs144645090 | A | G | -0.0082 | 0.0565 | 0.885 |
| rs147711004 | A | G | 0.1073 | 0.041 | 0.008789 |
| rs4802241 | C | A | #N/A | #N/A | #N/A |
| rs3112439 | C | G | #N/A | #N/A | #N/A |
| rs111278137 | A | G | -0.1624 | 0.0812 | 0.04561 |
| rs72654437 | A | G | -0.0152 | 0.0479 | 0.751 |

|  |  |  | **Coronary Heart Disease** | | |
| --- | --- | --- | --- | --- | --- |
| **SNP** | **effect allele** | **other allele** | **Beta.outcome** | **se.outcome** | **pval.outcome** |
| rs679515 | C | T | #N/A | #N/A | #N/A |
| rs6710467 | A | G | -0.03915 | 0.019293 | 4.24E-02 |
| rs6733839 | T | C | #N/A | #N/A | #N/A |
| rs9381563 | T | C | 0.016631 | 0.017383 | 3.39E-01 |
| rs114812713 | C | G | #N/A | #N/A | #N/A |
| rs34665982 | C | T | #N/A | #N/A | #N/A |
| rs11767557 | C | T | 0.01968 | 0.022068 | 3.73E-01 |
| rs73223431 | T | C | #N/A | #N/A | #N/A |
| rs867230 | A | C | #N/A | #N/A | #N/A |
| rs11257242 | G | C | #N/A | #N/A | #N/A |
| rs3740688 | T | G | #N/A | #N/A | #N/A |
| rs3851179 | C | T | 5.80E-03 | 1.44E-02 | 6.88E-01 |
| rs1582763 | A | G | 0.003985 | 0.014236 | 7.80E-01 |
| rs12590654 | A | G | #N/A | #N/A | #N/A |
| rs12151021 | G | A | #N/A | #N/A | #N/A |
| rs77301115 | A | G | #N/A | #N/A | #N/A |
| rs117316645 | A | G | #N/A | #N/A | #N/A |
| rs75463276 | G | C | #N/A | #N/A | #N/A |
| rs2965112 | G | A | #N/A | #N/A | #N/A |
| rs72654445 | A | G | #N/A | #N/A | #N/A |
| rs62117224 | A | G | #N/A | #N/A | #N/A |
| rs11669005 | A | G | #N/A | #N/A | #N/A |
| rs2965169 | C | A | #N/A | #N/A | #N/A |
| rs138607350 | G | T | #N/A | #N/A | #N/A |
| rs11668327 | C | G | #N/A | #N/A | #N/A |
| rs8100183 | T | C | #N/A | #N/A | #N/A |
| rs144645090 | A | G | #N/A | #N/A | #N/A |
| rs147711004 | A | G | #N/A | #N/A | #N/A |
| rs4802241 | C | A | #N/A | #N/A | #N/A |
| rs3112439 | C | G | #N/A | #N/A | #N/A |
| rs111278137 | A | G | #N/A | #N/A | #N/A |
| rs72654437 | A | G | #N/A | #N/A | #N/A |

|  |  |  | **Angina Pectoris** | | |
| --- | --- | --- | --- | --- | --- |
| **SNP** | **effect allele** | **other allele** | **Beta.outcome** | **se.outcome** | **pval.outcome** |
| rs679515 | C | T | -0.0116 | 0.0175 | 5.07E-01 |
| rs6710467 | A | G | -0.0012 | 0.0198 | 9.51E-01 |
| rs6733839 | T | C | -0.0071 | 0.0144 | 6.20E-01 |
| rs9381563 | T | C | 0.0064 | 0.0159 | 6.86E-01 |
| rs114812713 | C | G | #N/A | #N/A | #N/A |
| rs34665982 | C | T | #N/A | #N/A | #N/A |
| rs11767557 | C | T | -0.0162 | 0.0187 | 3.86E-01 |
| rs73223431 | T | C | -0.0142 | 0.0143 | 3.21E-01 |
| rs867230 | A | C | -0.0155 | 0.0141 | 2.71E-01 |
| rs11257242 | G | C | #N/A | #N/A | #N/A |
| rs3740688 | T | G | 0.0241 | 0.0156 | 1.22E-01 |
| rs3851179 | C | T | 0.0223 | 0.0144 | 1.21E-01 |
| rs1582763 | A | G | 0.0027 | 0.0155 | 8.61E-01 |
| rs12590654 | A | G | -0.022 | 0.0148 | 1.38E-01 |
| rs12151021 | G | A | -0.0321 | 0.0156 | 3.98E-02 |
| rs77301115 | A | G | 0.02 | 0.0473 | 6.72E-01 |
| rs117316645 | A | G | -0.0431 | 0.0367 | 2.41E-01 |
| rs75463276 | G | C | #N/A | #N/A | #N/A |
| rs2965112 | G | A | -0.0024 | 0.0221 | 9.15E-01 |
| rs72654445 | A | G | 0.1153 | 0.0664 | 8.27E-02 |
| rs62117224 | A | G | -0.0089 | 0.0145 | 5.39E-01 |
| rs11669005 | A | G | -0.0074 | 0.0146 | 6.10E-01 |
| rs2965169 | C | A | -0.0451 | 0.0145 | 1.84E-03 |
| rs138607350 | G | T | 0.0354 | 0.0497 | 4.77E-01 |
| rs11668327 | C | G | #N/A | #N/A | #N/A |
| rs8100183 | T | C | 0.0166 | 0.017 | 0.3288 |
| rs144645090 | A | G | 0.0714 | 0.0477 | 0.1346 |
| rs147711004 | A | G | 0.0961 | 0.0346 | 0.005439 |
| rs4802241 | C | A | #N/A | #N/A | #N/A |
| rs3112439 | C | G | #N/A | #N/A | #N/A |
| rs111278137 | A | G | -0.077 | 0.0672 | 0.2524 |
| rs72654437 | A | G | -0.0125 | 0.0405 | 0.7568 |

|  |  |  | **Ischemic Stroke** | | |
| --- | --- | --- | --- | --- | --- |
| **SNP** | **effect allele** | **other allele** | **Beta.outcome** | **se.outcome** | **pval.outcome** |
| rs679515 | C | T | -0.0143 | 0.0123 | 2.46E-01 |
| rs6710467 | A | G | -0.0027 | 0.0114 | 8.15E-01 |
| rs6733839 | T | C | -0.0088 | 0.009 | 3.31E-01 |
| rs9381563 | T | C | 0.0037 | 0.0091 | 6.81E-01 |
| rs114812713 | C | G | #N/A | #N/A | #N/A |
| rs34665982 | C | T | 0.0039 | 0.0123 | 7.52E-01 |
| rs11767557 | C | T | 0.0046 | 0.0107 | 6.69E-01 |
| rs73223431 | T | C | -0.0092 | 0.0089 | 3.00E-01 |
| rs867230 | A | C | -0.004 | 0.009 | 6.54E-01 |
| rs11257242 | G | C | #N/A | #N/A | #N/A |
| rs3740688 | T | G | -0.0138 | 0.0084 | 9.82E-02 |
| rs3851179 | C | T | 0.0121 | 0.0088 | 1.69E-01 |
| rs1582763 | A | G | 0.0173 | 0.009 | 5.65E-02 |
| rs12590654 | A | G | -0.0079 | 0.0095 | 4.07E-01 |
| rs12151021 | G | A | -0.0018 | 0.0093 | 8.45E-01 |
| rs77301115 | A | G | 0.0284 | 0.0327 | 3.85E-01 |
| rs117316645 | A | G | 0.0455 | 0.0241 | 5.91E-02 |
| rs75463276 | G | C | #N/A | #N/A | #N/A |
| rs2965112 | G | A | -0.0073 | 0.0131 | 5.79E-01 |
| rs72654445 | A | G | #N/A | #N/A | #N/A |
| rs62117224 | A | G | -0.0041 | 0.0132 | 7.58E-01 |
| rs11669005 | A | G | #N/A | #N/A | #N/A |
| rs2965169 | C | A | -0.0184 | 0.009 | 4.00E-02 |
| rs138607350 | G | T | #N/A | #N/A | #N/A |
| rs11668327 | C | G | #N/A | #N/A | #N/A |
| rs8100183 | T | C | 0.0057 | 0.0119 | 0.631899 |
| rs144645090 | A | G | -0.0145 | 0.0377 | 0.7001 |
| rs147711004 | A | G | 0.0068 | 0.0289 | 0.815 |
| rs4802241 | C | A | 0.0096 | 0.0113 | 0.3973 |
| rs3112439 | C | G | #N/A | #N/A | #N/A |
| rs111278137 | A | G | #N/A | #N/A | #N/A |
| rs72654437 | A | G | 0.0126 | 0.0334 | 0.707301 |

|  |  |  | **Large-artery Atherosclerotic Stroke** | | |
| --- | --- | --- | --- | --- | --- |
| **SNP** | **effect allele** | **other allele** | **Beta.outcome** | **se.outcome** | **pval.outcome** |
| rs679515 | C | T | -0.043 | 0.0299 | 1.51E-01 |
| rs6710467 | A | G | 0.0097 | 0.0273 | 7.23E-01 |
| rs6733839 | T | C | 0.0097 | 0.0215 | 6.51E-01 |
| rs9381563 | T | C | 0.025 | 0.0221 | 2.58E-01 |
| rs114812713 | C | G | #N/A | #N/A | #N/A |
| rs34665982 | C | T | -0.0385 | 0.0291 | 1.86E-01 |
| rs11767557 | C | T | -0.0146 | 0.0259 | 5.72E-01 |
| rs73223431 | T | C | 0.01 | 0.0215 | 6.41E-01 |
| rs867230 | A | C | -0.0192 | 0.0214 | 3.70E-01 |
| rs11257242 | G | C | #N/A | #N/A | #N/A |
| rs3740688 | T | G | -0.0041 | 0.0203 | 8.41E-01 |
| rs3851179 | C | T | 0.0127 | 0.0212 | 5.48E-01 |
| rs1582763 | A | G | 0.0132 | 0.0218 | 5.45E-01 |
| rs12590654 | A | G | -0.0393 | 0.0214 | 6.62E-02 |
| rs12151021 | G | A | 0.012 | 0.0222 | 5.90E-01 |
| rs77301115 | A | G | -0.1688 | 0.1091 | 1.22E-01 |
| rs117316645 | A | G | 0.0498 | 0.0664 | 4.53E-01 |
| rs75463276 | G | C | #N/A | #N/A | #N/A |
| rs2965112 | G | A | 0.0174 | 0.0313 | 5.77E-01 |
| rs72654445 | A | G | #N/A | #N/A | #N/A |
| rs62117224 | A | G | 0.0129 | 0.0312 | 6.80E-01 |
| rs11669005 | A | G | #N/A | #N/A | #N/A |
| rs2965169 | C | A | -0.0655 | 0.0218 | 2.65E-03 |
| rs138607350 | G | T | #N/A | #N/A | #N/A |
| rs11668327 | C | G | #N/A | #N/A | #N/A |
| rs8100183 | T | C | 0.0175 | 0.0291 | 0.5478 |
| rs144645090 | A | G | 0.0918 | 0.1033 | 0.3742 |
| rs147711004 | A | G | 0.1261 | 0.0762 | 0.097949 |
| rs4802241 | C | A | 0.005 | 0.0267 | 0.8512 |
| rs3112439 | C | G | #N/A | #N/A | #N/A |
| rs111278137 | A | G | 0.2541 | 0.1099 | 0.02077 |
| rs72654437 | A | G | -0.0861 | 0.0938 | 0.3587 |

|  |  |  | **Cardioembolic Stroke** | | |
| --- | --- | --- | --- | --- | --- |
| **SNP** | **effect allele** | **other allele** | **Beta.outcome** | **se.outcome** | **pval.outcome** |
| rs679515 | C | T | 0.0465 | 0.0247 | 6.02E-02 |
| rs6710467 | A | G | 0.0003 | 0.0237 | 9.89E-01 |
| rs6733839 | T | C | -0.0257 | 0.0182 | 1.59E-01 |
| rs9381563 | T | C | 0.0174 | 0.0184 | 3.44E-01 |
| rs114812713 | C | G | #N/A | #N/A | #N/A |
| rs34665982 | C | T | -0.0045 | 0.0226 | 8.43E-01 |
| rs11767557 | C | T | 0.0492 | 0.022 | 2.55E-02 |
| rs73223431 | T | C | 0.0006 | 0.018 | 9.74E-01 |
| rs867230 | A | C | -0.0139 | 0.0183 | 4.47E-01 |
| rs11257242 | G | C | #N/A | #N/A | #N/A |
| rs3740688 | T | G | -0.0099 | 0.0172 | 5.63E-01 |
| rs3851179 | C | T | -0.0071 | 0.0181 | 6.95E-01 |
| rs1582763 | A | G | 0.0166 | 0.0182 | 3.60E-01 |
| rs12590654 | A | G | -0.0042 | 0.0185 | 8.21E-01 |
| rs12151021 | G | A | -0.009 | 0.0191 | 6.37E-01 |
| rs77301115 | A | G | -0.1738 | 0.0806 | 3.10E-02 |
| rs117316645 | A | G | 0.1008 | 0.0499 | 4.36E-02 |
| rs75463276 | G | C | #N/A | #N/A | #N/A |
| rs2965112 | G | A | 0.0101 | 0.027 | 7.09E-01 |
| rs72654445 | A | G | #N/A | #N/A | #N/A |
| rs62117224 | A | G | -0.0077 | 0.0234 | 7.42E-01 |
| rs11669005 | A | G | #N/A | #N/A | #N/A |
| rs2965169 | C | A | -0.0065 | 0.0184 | 7.24E-01 |
| rs138607350 | G | T | #N/A | #N/A | #N/A |
| rs11668327 | C | G | #N/A | #N/A | #N/A |
| rs8100183 | T | C | 0.0225 | 0.0245 | 0.3565 |
| rs144645090 | A | G | -0.0298 | 0.0855 | 0.7274 |
| rs147711004 | A | G | 0.0034 | 0.0594 | 0.9544 |
| rs4802241 | C | A | -0.0238 | 0.0241 | 0.322 |
| rs3112439 | C | G | #N/A | #N/A | #N/A |
| rs111278137 | A | G | 0.2115 | 0.088 | 0.01624 |
| rs72654437 | A | G | 0.006 | 0.0663 | 0.9279 |

# effect_allele= effect alleles; other_allele=minor allele; beta.exposure=effect size of SNP on exposure; beta.outcome=effect size of SNP on outcome; pval.outcome=p-value for outcome; pval.exposure=p-value for exposure; se.outcome=standard error for outcome; se.exposure=standard error for exposure; eaf.exposure=Effect allele frequency for exposure; R2= the proportion of variance in the exposure explained by the genetic variants; F= F-statistic,F-statistic greater than or equal to 10 indicates a relatively low risk of weak instrument bias in MR analysis.#N/A stands for actual SNP information.
