# Supplementary material for: Causal relationship between Alzheimer’s disease and cardiovascular disease: a bidirectional Mendelian randomization analysis
Source: Aging (Albany NY). 2023 Sep 2;15(17):9022–40. doi: 10.18632/aging.205013 (PMC10522384; doi:10.18632/aging.205013)
Supplement: Supplementary Table 2 [file aging-15-205013-s003.docx]

**Supplementary Table 2. SNPs information of cardiovascular disease with Alzheimer's disease.**

|  | |  | |  | | **Atrial Fibrillation** | | | | | | **Alzheimer's Disease** | | | | | |
| --- | --- | --- | --- | --- | --- | --- | --- | --- | --- | --- | --- | --- | --- | --- | --- | --- | --- |
| **SNP** | | **effect allele** | | **other allele** | | **Beta.**  **exposure** | **Se.**  **exposure** | | **Pval.**  **exposure** | **R2** | **F** | **Beta.**  **outcome** | | **Se.**  **outcome** | | **Pval.**  **outcome** | |
| rs10786662 | | C | | G | | -0.051 | 0.008 | | 1.99E-11 | 3.34E-03 | 44.672 | #N/A | | #N/A | | #N/A | |
| rs11492754 | | T | | C | | 0.060 | 0.009 | | 1.41E-11 | 2.69E-03 | 46.019 | -0.014 | | 0.017 | | 0.414 | |
| rs11598047 | | A | | G | | -0.146 | 0.010 | | 1.02E-47 | 1.21E-02 | 211.97 | 0.0105 | | 0.019 | | 0.583 | |
| rs41287510 | | A | | G | | -0.125 | 0.020 | | 8.44E-10 | 2.17E-03 | 37.67 | 0.0363 | | 0.04 | | 0.367 | |
| rs35176054 | | A | | T | | 0.148 | 0.011 | | 1.04E-40 | 9.97E-03 | 178 | #N/A | | #N/A | | #N/A | |
| rs7919685 | | T | | G | | -0.058 | 0.008 | | 1.62E-14 | 1.80E-04 | 59.804 | 0.0054 | | 0.014 | | 0.705 | |
| rs6480708 | | A | | C | | -0.115 | 0.011 | | 5.79E-27 | 3.00E-04 | 116.32 | #N/A | | #N/A | | #N/A | |
| rs11001667 | | A | | G | | -0.055 | 0.010 | | 3.84E-08 | 7.70E-05 | 30.14 | 0.0215 | | 0.019 | | 0.252 | |
| rs949078 | | T | | C | | -0.049 | 0.009 | | 2.09E-08 | 5.70E-05 | 31.721 | 0.0226 | | 0.017 | | 0.171 | |
| rs76097649 | | A | | G | | 0.131 | 0.014 | | 9.28E-21 | 1.50E-04 | 86.846 | 0.0198 | | 0.033 | | 0.546 | |
| rs2625322 | | A | | G | | 0.069 | 0.009 | | 1.78E-14 | 9.20E-05 | 58.607 | -0.031 | | 0.017 | | 0.07 | |
| rs883079 | | T | | C | | 0.115 | 0.009 | | 4.81E-41 | 1.80E-04 | 177.88 | #N/A | | #N/A | | #N/A | |
| rs11835327 | | A | | G | | -0.075 | 0.013 | | 1.16E-08 | 3.30E-05 | 32.516 | -0.02 | | 0.025 | | 0.429 | |
| rs12810346 | | T | | C | | 0.064 | 0.011 | | 8.63E-09 | 3.30E-05 | 33.037 | 0.0161 | | 0.022 | | 0.467 | |
| rs3789967 | | T | | G | | 0.046 | 0.008 | | 5.93E-09 | 3.20E-05 | 33.495 | #N/A | | #N/A | | #N/A | |
| rs6487400 | | T | | C | | -0.057 | 0.010 | | 8.32E-09 | 2.90E-05 | 32.948 | -0.012 | | 0.019 | | 0.527 | |
| rs10842383 | | T | | C | | -0.104 | 0.011 | | 1.11E-21 | 8.10E-05 | 90.861 | #N/A | | #N/A | | #N/A | |
| rs113819537 | | C | | G | | 0.051 | 0.009 | | 6.55E-09 | 3.00E-05 | 33.324 | #N/A | | #N/A | | #N/A | |
| rs12809354 | | T | | C | | -0.086 | 0.011 | | 2.79E-16 | 5.80E-05 | 66.617 | 0.0046 | | 0.02 | | 0.82 | |
| rs2860482 | | A | | C | | 0.051 | 0.009 | | 1.25E-09 | 3.00E-05 | 36.567 | -1E-04 | | 0.016 | | 0.997 | |
| rs35349325 | | T | | C | | 0.055 | 0.008 | | 7.83E-13 | 4.00E-05 | 51.424 | -0.002 | | 0.015 | | 0.892 | |
| rs11180703 | | A | | G | | -0.044 | 0.008 | | 7.62E-09 | 2.60E-05 | 33.366 | -0.016 | | 0.014 | | 0.268 | |
| rs7987944 | | T | | C | | -0.057 | 0.008 | | 1.37E-12 | 3.40E-05 | 49.879 | -0.02 | | 0.015 | | 0.18 | |
| rs28631169 | | T | | C | | 0.074 | 0.010 | | 6.65E-15 | 3.40E-05 | 60.348 | -0.016 | | 0.018 | | 0.39 | |
| rs1957021 | | T | | C | | -0.066 | 0.009 | | 1.31E-13 | 3.00E-05 | 54.993 | 0.0146 | | 0.017 | | 0.391 | |
| rs2145587 | | A | | G | | 0.072 | 0.009 | | 2.47E-17 | 4.00E-05 | 72.15 | #N/A | | #N/A | | #N/A | |
| rs11846704 | | T | | C | | 0.060 | 0.009 | | 9.40E-13 | 2.80E-05 | 50.494 | -0.022 | | 0.016 | | 0.18 | |
| rs2738413 | | A | | G | | 0.078 | 0.008 | | 1.06E-24 | 5.50E-05 | 104.79 | 0.0042 | | 0.015 | | 0.774 | |
| rs8181996 | | C | | G | | -0.047 | 0.008 | | 2.48E-09 | 1.80E-05 | 35.848 | #N/A | | #N/A | | #N/A | |
| rs146311723 | | T | | C | | -0.057 | 0.010 | | 6.17E-09 | 1.50E-05 | 33.83 | #N/A | | #N/A | | #N/A | |
| rs12591736 | | A | | G | | -0.063 | 0.011 | | 2.82E-08 | 1.40E-05 | 30.69 | 0.0117 | | 0.021 | | 0.583 | |
| rs7172038 | | T | | G | | -0.108 | 0.010 | | 2.30E-27 | 5.30E-05 | 116.86 | -0.022 | | 0.02 | | 0.253 | |
| rs12908004 | | A | | G | | -0.075 | 0.010 | | 1.92E-13 | 2.40E-05 | 54.354 | -0.012 | | 0.02 | | 0.552 | |
| rs12908437 | | T | | C | | 0.050 | 0.008 | | 2.12E-10 | 1.70E-05 | 40.274 | 0.0113 | | 0.015 | | 0.455 | |
| rs30252 | | T | | C | | 0.065 | 0.009 | | 3.79E-14 | 2.40E-05 | 57.478 | -0.002 | | 0.017 | | 0.895 | |
| rs77316573 | | T | | C | | 0.064 | 0.010 | | 3.23E-10 | 1.70E-05 | 39.777 | #N/A | | #N/A | | #N/A | |
| rs2359171 | | A | | T | | 0.179 | 0.010 | | 1.51E-76 | 1.40E-04 | 340.92 | #N/A | | #N/A | | #N/A | |
| rs76774446 | | A | | C | | 0.065 | 0.011 | | 4.10E-09 | 1.30E-05 | 34.714 | -0.012 | | 0.022 | | 0.593 | |
| rs3844438 | | T | | G | | 0.045 | 0.008 | | 2.62E-09 | 1.30E-05 | 35.84 | -0.022 | | 0.014 | | 0.121 | |
| rs9675122 | | C | | G | | 0.054 | 0.008 | | 4.78E-12 | 1.70E-05 | 47.418 | #N/A | | #N/A | | #N/A | |
| rs9953366 | | T | | C | | -0.053 | 0.008 | | 2.00E-10 | 1.40E-05 | 40.468 | -0.022 | | 0.016 | | 0.166 | |
| rs1443926 | | A | | G | | 0.048 | 0.008 | | 4.00E-09 | 1.00E-05 | 34.265 | -0.011 | | 0.015 | | 0.485 | |
| rs4484922 | | C | | G | | -0.066 | 0.008 | | 2.73E-15 | 1.80E-05 | 62.297 | #N/A | | #N/A | | #N/A | |
| rs79187193 | | A | | G | | -0.117 | 0.018 | | 2.44E-10 | 1.20E-05 | 40.157 | -0.059 | | 0.032 | | 0.067 | |
| rs7549338 | | C | | G | | 0.046 | 0.008 | | 1.02E-09 | 1.10E-05 | 37.274 | #N/A | | #N/A | | #N/A | |
| rs4999127 | | A | | G | | 0.090 | 0.011 | | 6.09E-16 | 1.90E-05 | 65.595 | -0.013 | | 0.021 | | 0.543 | |
| rs1218574 | | A | | G | | -0.078 | 0.011 | | 4.62E-12 | 1.40E-05 | 47.647 | 0.018 | | 0.021 | | 0.384 | |
| rs11264280 | | T | | C | | 0.129 | 0.008 | | 1.05E-57 | 7.40E-05 | 254.03 | -0.009 | | 0.016 | | 0.572 | |
| rs72700114 | | C | | G | | 0.206 | 0.014 | | 1.03E-46 | 5.90E-05 | 206.51 | #N/A | | #N/A | | #N/A | |
| rs12122060 | | A | | T | | 0.137 | 0.011 | | 8.47E-34 | 4.20E-05 | 146.77 | #N/A | | #N/A | | #N/A | |
| rs503706 | | T | | C | | 0.100 | 0.008 | | 1.87E-39 | 4.90E-05 | 172.09 | 0.0054 | | 0.015 | | 0.709 | |
| rs12121494 | | A | | G | | 0.206 | 0.037 | | 2.11E-08 | 9.00E-06 | 31.384 | 0.0348 | | 0.07 | | 0.618 | |
| rs4590732 | | C | | G | | 0.067 | 0.008 | | 1.07E-18 | 2.20E-05 | 77.95 | #N/A | | #N/A | | #N/A | |
| rs951366 | | T | | C | | -0.045 | 0.008 | | 8.18E-09 | 9.20E-06 | 33.399 | -0.015 | | 0.015 | | 0.294 | |
| rs146518726 | | A | | G | | 0.162 | 0.025 | | 2.05E-10 | 1.00E-05 | 40.528 | -0.014 | | 0.047 | | 0.761 | |
| rs7269123 | | T | | C | | -0.045 | 0.008 | | 2.94E-08 | 7.30E-06 | 30.727 | #N/A | | #N/A | | #N/A | |
| rs2145274 | | A | | C | | 0.105 | 0.015 | | 5.36E-13 | 1.20E-05 | 51.722 | -0.016 | | 0.025 | | 0.523 | |
| rs2834618 | | T | | G | | 0.107 | 0.013 | | 4.64E-16 | 1.50E-05 | 65.831 | -0.004 | | 0.023 | | 0.866 | |
| rs464901 | | T | | C | | 0.054 | 0.008 | | 8.67E-11 | 9.60E-06 | 42.015 | 0.0217 | | 0.015 | | 0.153 | |
| rs56181519 | | T | | C | | -0.081 | 0.009 | | 6.14E-20 | 1.80E-05 | 84.306 | 0.0097 | | 0.017 | | 0.562 | |
| rs2288327 | | A | | G | | -0.100 | 0.010 | | 3.48E-23 | 2.00E-05 | 97.834 | -0.029 | | 0.019 | | 0.13 | |
| rs295114 | | T | | C | | -0.065 | 0.008 | | 3.98E-17 | 1.40E-05 | 70.386 | 0.0072 | | 0.014 | | 0.617 | |
| rs7578393 | | T | | C | | 0.069 | 0.010 | | 5.83E-12 | 9.40E-06 | 47.334 | -0.027 | | 0.019 | | 0.16 | |
| rs2441380 | | T | | C | | -0.047 | 0.008 | | 1.77E-09 | 6.90E-06 | 36 | -0.006 | | 0.015 | | 0.682 | |
| rs2723064 | | T | | C | | 0.071 | 0.008 | | 7.79E-20 | 1.60E-05 | 82.857 | 0.0194 | | 0.015 | | 0.185 | |
| rs6546553 | | A | | C | | -0.065 | 0.008 | | 1.21E-17 | 1.40E-05 | 73.598 | -0.021 | | 0.015 | | 0.139 | |
| rs72926475 | | A | | G | | -0.077 | 0.012 | | 4.04E-11 | 8.20E-06 | 43.492 | -0.011 | | 0.021 | | 0.603 | |
| rs73228543 | | A | | G | | -0.072 | 0.011 | | 3.02E-11 | 8.20E-06 | 43.997 | -0.002 | | 0.021 | | 0.943 | |
| rs7650482 | | A | | G | | -0.077 | 0.008 | | 4.46E-22 | 1.70E-05 | 94.508 | -0.018 | | 0.015 | | 0.238 | |
| rs4855074 | | T | | C | | 0.064 | 0.011 | | 3.07E-09 | 6.20E-06 | 34.907 | 0.0181 | | 0.021 | | 0.377 | |
| rs9872035 | | T | | C | | -0.043 | 0.008 | | 1.80E-08 | 5.60E-06 | 31.567 | -0.018 | | 0.014 | | 0.197 | |
| rs41312411 | | C | | G | | 0.064 | 0.011 | | 3.16E-09 | 6.20E-06 | 35.441 | #N/A | | #N/A | | #N/A | |
| rs7373065 | | T | | C | | 0.215 | 0.029 | | 6.50E-14 | 9.80E-06 | 56.172 | -0.04 | | 0.053 | | 0.443 | |
| rs6790396 | | C | | G | | -0.060 | 0.008 | | 5.73E-15 | 1.10E-05 | 60.516 | #N/A | | #N/A | | #N/A | |
| rs332388 | | T | | C | | -0.047 | 0.008 | | 7.74E-10 | 6.40E-06 | 37.416 | 0.004 | | 0.015 | | 0.783 | |
| rs7632427 | | T | | C | | 0.046 | 0.008 | | 2.43E-09 | 6.00E-06 | 35.689 | #N/A | | #N/A | | #N/A | |
| rs11727818 | | A | | G | | -0.045 | 0.008 | | 3.99E-08 | 5.10E-06 | 30.25 | 0.0145 | | 0.016 | | 0.368 | |
| rs3960788 | | T | | C | | -0.051 | 0.008 | | 4.48E-11 | 7.20E-06 | 43.013 | -9E-04 | | 0.015 | | 0.952 | |
| rs1963560 | | T | | C | | 0.067 | 0.011 | | 5.41E-10 | 6.40E-06 | 38.742 | 0.0082 | | 0.021 | | 0.694 | |
| rs17041835 | | A | | G | | 0.121 | 0.021 | | 1.29E-08 | 5.40E-06 | 32.522 | 0.0307 | | 0.047 | | 0.508 | |
| rs72666200 | | T | | C | | -0.092 | 0.011 | | 1.54E-16 | 1.10E-05 | 67.621 | 0.0074 | | 0.021 | | 0.724 | |
| rs1470618 | | T | | C | | 0.131 | 0.010 | | 1.62E-39 | 2.90E-05 | 174.83 | 0.0115 | | 0.021 | | 0.584 | |
| rs17746631 | | A | | G | | 0.165 | 0.018 | | 3.29E-19 | 1.30E-05 | 80.609 | 0.0184 | | 0.033 | | 0.578 | |
| rs6854883 | | T | | C | | 0.326 | 0.010 | | ###### | 1.80E-04 | 1081 | -0.027 | | 0.02 | | 0.174 | |
| rs62337205 | | A | | G | | 0.252 | 0.021 | | 9.86E-33 | 2.40E-05 | 141.63 | -0.002 | | 0.051 | | 0.967 | |
| rs115782574 | | A | | G | | 0.215 | 0.029 | | 5.21E-14 | 9.40E-06 | 56.645 | -0.044 | | 0.06 | | 0.459 | |
| rs28601812 | | A | | C | | 0.079 | 0.009 | | 2.18E-19 | 1.30E-05 | 81.205 | 0.0198 | | 0.017 | | 0.23 | |
| rs3853445 | | T | | C | | 0.200 | 0.009 | | ###### | 8.60E-05 | 515.5 | -0.015 | | 0.016 | | 0.338 | |
| rs416532 | | T | | G | | 0.088 | 0.008 | | 7.08E-28 | 2.00E-05 | 119.63 | -0.004 | | 0.015 | | 0.808 | |
| rs149496090 | | A | | T | | -0.262 | 0.046 | | 1.43E-08 | 5.30E-06 | 32.111 | #N/A | | #N/A | | #N/A | |
| rs449333 | | C | | G | | 0.078 | 0.008 | | 3.25E-21 | 1.50E-05 | 89.326 | #N/A | | #N/A | | #N/A | |
| rs55754224 | | T | | C | | 0.051 | 0.009 | | 2.48E-09 | 6.00E-06 | 36 | -0.016 | | 0.016 | | 0.316 | |
| rs10027347 | | T | | G | | 0.112 | 0.016 | | 7.41E-13 | 8.40E-06 | 51.637 | -0.023 | | 0.03 | | 0.456 | |
| rs10520260 | | A | | G | | 0.051 | 0.008 | | 8.03E-10 | 6.10E-06 | 37.904 | 0.0016 | | 0.016 | | 0.918 | |
| rs78243103 | | A | | G | | 0.149 | 0.025 | | 3.90E-09 | 5.50E-06 | 34.684 | -0.033 | | 0.051 | | 0.516 | |
| rs716845 | | A | | G | | 0.054 | 0.008 | | 1.33E-10 | 6.20E-06 | 41.548 | 0.0109 | | 0.016 | | 0.488 | |
| rs10479177 | | A | | G | | -0.096 | 0.008 | | 2.03E-32 | 2.10E-05 | 139.59 | 0.0119 | | 0.016 | | 0.445 | |
| rs174048 | | T | | C | | -0.067 | 0.010 | | 5.74E-11 | 6.30E-06 | 42.761 | -0.016 | | 0.02 | | 0.433 | |
| rs6891790 | | T | | G | | -0.066 | 0.009 | | 1.10E-14 | 8.60E-06 | 59.433 | -0.01 | | 0.017 | | 0.563 | |
| rs210632 | | A | | G | | 0.048 | 0.009 | | 2.75E-08 | 4.20E-06 | 30.764 | -0.004 | | 0.016 | | 0.796 | |
| rs9481842 | | T | | G | | -0.069 | 0.008 | | 1.84E-16 | 9.30E-06 | 67.866 | 0.0197 | | 0.016 | | 0.217 | |
| rs868155 | | T | | C | | -0.065 | 0.008 | | 2.31E-16 | 9.30E-06 | 68.115 | -0.02 | | 0.015 | | 0.18 | |
| rs117984853 | | T | | G | | 0.115 | 0.014 | | 3.14E-17 | 9.60E-06 | 71.751 | 0.0197 | | 0.029 | | 0.502 | |
| rs7770062 | | A | | G | | -0.107 | 0.011 | | 9.11E-21 | 1.20E-05 | 87.932 | -0.011 | | 0.021 | | 0.6 | |
| rs34969716 | | A | | G | | 0.084 | 0.009 | | 3.94E-22 | 1.20E-05 | 93.222 | 0.0089 | | 0.018 | | 0.627 | |
| rs762624 | | A | | C | | 0.057 | 0.009 | | 7.15E-11 | 5.50E-06 | 42.325 | -0.006 | | 0.016 | | 0.737 | |
| rs11773845 | | A | | C | | 0.115 | 0.008 | | 1.57E-50 | 2.80E-05 | 223.06 | #N/A | | #N/A | | #N/A | |
| rs55985730 | | T | | G | | -0.096 | 0.017 | | 2.00E-08 | 4.00E-06 | 31.558 | -0.035 | | 0.034 | | 0.299 | |
| rs55734480 | | A | | G | | 0.056 | 0.009 | | 1.23E-10 | 5.20E-06 | 41.58 | -0.016 | | 0.017 | | 0.35 | |
| rs7789146 | | A | | G | | -0.066 | 0.010 | | 8.57E-11 | 5.20E-06 | 42.186 | 0.0318 | | 0.019 | | 0.098 | |
| rs6462078 | | A | | C | | 0.058 | 0.009 | | 3.12E-11 | 5.40E-06 | 43.74 | 0.0367 | | 0.017 | | 0.026 | |
| rs74910854 | | A | | G | | -0.090 | 0.016 | | 4.27E-08 | 3.60E-06 | 30.049 | 0.0278 | | 0.035 | | 0.431 | |
| rs11773884 | | A | | G | | 0.049 | 0.008 | | 4.72E-09 | 4.10E-06 | 34.286 | #N/A | | #N/A | | #N/A | |
| rs78332318 | | T | | C | | 0.132 | 0.016 | | 9.35E-17 | 8.10E-06 | 68.817 | -0.002 | | 0.031 | | 0.941 | |
| rs4871397 | | C | | G | | -0.100 | 0.016 | | 1.07E-10 | 4.90E-06 | 41.79 | #N/A | | #N/A | | #N/A | |
| rs35006907 | | A | | C | | 0.048 | 0.008 | | 2.82E-09 | 4.20E-06 | 35.557 | -0.004 | | 0.016 | | 0.818 | |
| rs7460121 | | A | | G | | 0.072 | 0.013 | | 2.15E-08 | 3.70E-06 | 31.29 | -0.022 | | 0.025 | | 0.38 | |
| rs4355822 | | A | | G | | 0.049 | 0.008 | | 1.60E-10 | 4.70E-06 | 40.725 | 0.0011 | | 0.014 | | 0.94 | |
| rs7508 | | A | | G | | 0.074 | 0.009 | | 4.16E-18 | 8.80E-06 | 75.997 | -0.003 | | 0.016 | | 0.85 | |
| rs6998692 | | T | | C | | 0.087 | 0.012 | | 3.51E-13 | 6.10E-06 | 52.683 | #N/A | | #N/A | | #N/A | |
| rs7026071 | | T | | C | | 0.095 | 0.008 | | 1.77E-35 | 1.60E-05 | 152.86 | 0.0152 | | 0.015 | | 0.294 | |
|  | |  | |  | | **Heart Failure** | | | | | | **Alzheimer's Disease** | | | | | |
| **SNP** | | **effect allele** | | **other allele** | | **Beta.**  **exposure** | **Se.**  **exposure** | | **Pval.**  **exposure** | **R2** | **F** | **Beta.**  **outcome** | | **Se.**  **outcome** | | **Pval.**  **outcome** | |
| rs1556516 | | C | | G | | 0.062 | 0.008 | | 1.57E-15 | 1.70E-04 | 63.59 | #N/A | | #N/A | | #N/A | |
| rs17617337 | | T | | C | | -0.056 | 0.010 | | 3.65E-09 | 9.20E-05 | 34.872 | 0.0164 | | 0.018 | | 0.348 | |
| rs17042102 | | A | | G | | 0.110 | 0.012 | | 5.71E-20 | 7.50E-05 | 83.096 | -0.04 | | 0.022 | | 0.072 | |
| rs4746140 | | C | | G | | -0.067 | 0.011 | | 1.10E-09 | 2.60E-05 | 37.333 | #N/A | | #N/A | | #N/A | |
| rs600038 | | T | | C | | -0.057 | 0.010 | | 3.68E-09 | 2.10E-05 | 35.13 | 0.0135 | | 0.017 | | 0.436 | |
| rs4766578 | | A | | T | | -0.043 | 0.008 | | 4.90E-08 | 1.00E-05 | 30.041 | #N/A | | #N/A | | #N/A | |
| rs56094641 | | A | | G | | -0.045 | 0.008 | | 1.21E-08 | 1.10E-05 | 32.206 | -0.003 | | 0.015 | | 0.857 | |
| rs55730499 | | T | | C | | 0.106 | 0.016 | | 1.83E-11 | 1.00E-05 | 45.412 | -0.036 | | 0.033 | | 0.271 | |
| rs11745324 | | A | | G | | -0.053 | 0.010 | | 2.35E-08 | 6.40E-06 | 30.89 | -0.014 | | 0.017 | | 0.413 | |
| rs4135240 | | T | | C | | 0.049 | 0.008 | | 6.84E-09 | 6.90E-06 | 33.474 | -0.017 | | 0.015 | | 0.278 | |
| rs660240 | | T | | C | | -0.061 | 0.010 | | 3.25E-10 | 7.30E-06 | 39.677 | -0.005 | | 0.017 | | 0.753 | |
| rs9295128 | | T | | G | | 0.210 | 0.034 | | 4.96E-10 | 6.20E-06 | 38.712 | -0.017 | | 0.073 | | 0.821 | |
|  | |  | |  | | **Myocardial Infarction** | | | | | | **Alzheimer's Disease** | | | | | |
| **SNP** | | **effect allele** | | **other allele** | | **Beta.**  **exposure** | **Se.**  **exposure** | | **Pval.**  **exposure** | **R2** | **F** | **Beta.**  **outcome** | | **Se.**  **outcome** | | **Pval.**  **outcome** | |
| rs4927191 | | C | | T | | -0.12 | 0.02 | | 7.86E-11 | 2.10E-04 | 42.25 | #N/A | | #N/A | | #N/A | |
| rs17414716 | | G | | A | | -0.27 | 0.05 | | 2.16E-08 | 1.60E-04 | 31.36 | 0.1283 | | 0.087 | | 0.14 | |
| rs72664324 | | A | | G | | -0.15 | 0.03 | | 1.58E-08 | 1.60E-04 | 32.038 | 0.0145 | | 0.025 | | 0.56 | |
| rs17612693 | | A | | T | | 0.162 | 0.02 | | 2.94E-11 | 2.20E-04 | 44.298 | #N/A | | #N/A | | #N/A | |
| rs4632638 | | A | | G | | -0.12 | 0.02 | | 2.97E-09 | 1.80E-04 | 35.16 | #N/A | | #N/A | | #N/A | |
| rs74617384 | | T | | A | | 0.337 | 0.04 | | 1.54E-17 | 3.60E-04 | 72.616 | #N/A | | #N/A | | #N/A | |
| rs9349379 | | G | | A | | 0.171 | 0.02 | | 7.60E-26 | 5.60E-04 | 111.16 | 0.0067 | | 0.015 | | 0.65 | |
| rs3918226 | | T | | C | | 0.183 | 0.03 | | 9.23E-09 | 1.70E-04 | 33.053 | -0.034 | | 0.028 | | 0.236 | |
| rs9644861 | | T | | C | | 0.235 | 0.02 | | 1.89E-46 | 1.03E-03 | 205.15 | #N/A | | #N/A | | #N/A | |
| rs137943060 | | A | | G | | 0.338 | 0.06 | | 2.63E-08 | 1.60E-04 | 30.951 | 0.025 | | 0.048 | | 0.604 | |
| rs117951873 | | T | | C | | -0.32 | 0.05 | | 1.48E-10 | 2.10E-04 | 41.058 | #N/A | | #N/A | | #N/A | |
| rs4887085 | | C | | G | | -0.11 | 0.02 | | 6.35E-10 | 1.90E-04 | 38.353 | #N/A | | #N/A | | #N/A | |
| rs12052058 | | T | | G | | -0.13 | 0.02 | | 7.41E-11 | 2.10E-04 | 42.413 | -0.015 | | 0.017 | | 0.371 | |
| rs7412 | | T | | C | | -0.26 | 0.04 | | 1.33E-12 | 2.50E-04 | 50.288 | -0.467 | | 0.031 | | 6E-53 | |
| rs28451064 | | A | | G | | 0.185 | 0.02 | | 2.75E-16 | 3.30E-04 | 66.706 | -0.02 | | 0.023 | | 0.383 | |
|  | |  | |  | | **Coronary Heart Disease** | | | | | | **Alzheimer's Disease** | | | | | |
| **SNP** | | **effect allele** | | **other allele** | | **Beta.**  **exposure** | **Se.**  **exposure** | | **Pval.**  **exposure** | **R2** | **F** | **Beta.**  **outcome** | | **Se.**  **outcome** | | **Pval.**  **outcome** | |
| rs599839 | | A | | G | | 0.107 | 0.017 | | 2.89E-10 | 4.70E-04 | 39.75 | 0.0093 | | 0.017 | | 0.588 | |
| rs17114036 | | G | | A | | -0.145 | 0.026 | | 1.43E-08 | 4.00E-04 | 32.144 | 0.0165 | | 0.025 | | 0.508 | |
| rs2351524 | | C | | T | | -0.139 | 0.021 | | 1.76E-11 | 5.40E-04 | 45.225 | 0.0538 | | 0.021 | | 0.012 | |
| rs2306374 | | C | | T | | 0.108 | 0.020 | | 3.34E-08 | 3.90E-04 | 30.497 | -0.027 | | 0.02 | | 0.165 | |
| rs7651039 | | C | | T | | 0.142 | 0.025 | | 1.85E-08 | 6.40E-04 | 31.646 | -0.01 | | 0.014 | | 0.471 | |
| rs10455872 | | G | | A | | 0.278 | 0.038 | | 3.08E-13 | 8.10E-04 | 53.155 | -0.036 | | 0.033 | | 0.269 | |
| rs12190287 | | G | | C | | -0.103 | 0.016 | | 4.64E-11 | 5.50E-04 | 43.32 | #N/A | | #N/A | | #N/A | |
| rs9351814 | | C | | A | | -0.080 | 0.014 | | 2.02E-08 | 3.80E-04 | 31.47 | 0.0052 | | 0.015 | | 0.723 | |
| rs4714955 | | T | | C | | -0.100 | 0.015 | | 6.03E-12 | 5.70E-04 | 47.32 | -0.002 | | 0.015 | | 0.885 | |
| rs11556924 | | T | | C | | -0.091 | 0.015 | | 2.22E-09 | 4.50E-04 | 35.773 | -0.002 | | 0.016 | | 0.889 | |
| rs1333045 | | C | | T | | 0.226 | 0.019 | | 4.63E-32 | 1.81E-03 | 138.9 | 0.0299 | | 0.015 | | 0.053 | |
| rs7127880 | | T | | C | | -0.238 | 0.039 | | 1.05E-09 | 4.50E-04 | 37.233 | #N/A | | #N/A | | #N/A | |
| rs964184 | | C | | G | | -0.126 | 0.021 | | 8.02E-10 | 4.60E-04 | 37.756 | #N/A | | #N/A | | #N/A | |
| rs2219939 | | A | | G | | -0.099 | 0.016 | | 1.21E-09 | 4.60E-04 | 36.959 | -0.002 | | 0.018 | | 0.908 | |
| rs1122608 | | T | | G | | -0.127 | 0.021 | | 9.73E-10 | 4.50E-04 | 37.377 | -0.016 | | 0.017 | | 0.321 | |
| rs9982601 | | T | | C | | 0.164 | 0.026 | | 4.22E-10 | 4.80E-04 | 39.008 | -0.026 | | 0.022 | | 0.235 | |
|  | |  | |  | | **Angina Pectoris** | | | | | | **Alzheimer's Disease** | | | | | |
| **SNP** | | **effect allele** | | **other allele** | | **Beta.**  **exposure** | **Se.**  **exposure** | | **Pval.**  **exposure** | **R2** | **F** | **Beta.**  **outcome** | | **Se.**  **outcome** | | **Pval.**  **outcome** | |
| rs646776 | | T | | C | | 0.093 | 0.017 | | 3.45E-08 | 1.50E-04 | 30.512 | 0.0089 | | 0.017 | | 0.601 | |
| rs11591147 | | T | | G | | -0.249 | 0.037 | | 2.37E-11 | 2.20E-04 | 44.599 | 0.0304 | | 0.078 | | 0.695 | |
| rs9970807 | | T | | C | | -0.132 | 0.022 | | 2.53E-09 | 1.70E-04 | 35.567 | 0.0173 | | 0.025 | | 0.483 | |
| rs7608755 | | A | | G | | 0.121 | 0.022 | | 2.56E-08 | 1.50E-04 | 30.96 | -0.052 | | 0.021 | | 0.014 | |
| rs7668383 | | C | | T | | -0.102 | 0.018 | | 1.96E-08 | 1.50E-04 | 31.633 | 0.0026 | | 0.019 | | 0.892 | |
| rs4835377 | | G | | A | | -0.092 | 0.017 | | 2.82E-08 | 1.50E-04 | 30.849 | 0.044 | | 0.019 | | 0.022 | |
| rs17612693 | | A | | T | | 0.155 | 0.021 | | 4.08E-14 | 2.80E-04 | 57.315 | #N/A | | #N/A | | #N/A | |
| rs1261370 | | A | | T | | -0.103 | 0.018 | | 2.02E-08 | 1.50E-04 | 31.433 | #N/A | | #N/A | | #N/A | |
| rs10455872 | | G | | A | | 0.347 | 0.033 | | 7.26E-26 | 5.40E-04 | 110.76 | -0.036 | | 0.033 | | 0.269 | |
| rs9349379 | | G | | A | | 0.114 | 0.014 | | 1.77E-16 | 3.30E-04 | 67.763 | 0.0067 | | 0.015 | | 0.65 | |
| rs117733303 | | G | | A | | 0.467 | 0.067 | | 2.49E-12 | 2.40E-04 | 49.042 | -0.032 | | 0.058 | | 0.579 | |
| rs12705390 | | A | | G | | 0.088 | 0.015 | | 3.29E-09 | 1.70E-04 | 35.199 | 0.0007 | | 0.018 | | 0.968 | |
| rs149714437 | | A | | G | | 0.121 | 0.022 | | 4.95E-08 | 1.40E-04 | 29.636 | 0.015 | | 0.047 | | 0.75 | |
| rs10757272 | | T | | C | | 0.205 | 0.014 | | 8.31E-49 | 1.05E-03 | 216.45 | 0.0319 | | 0.015 | | 0.036 | |
| rs117140212 | | A | | G | | 0.329 | 0.054 | | 1.20E-09 | 1.80E-04 | 36.915 | -0.019 | | 0.104 | | 0.854 | |
| rs2106116 | | T | | C | | 0.102 | 0.017 | | 1.16E-09 | 1.80E-04 | 37.152 | 0.0019 | | 0.018 | | 0.913 | |
| rs964184 | | C | | G | | -0.108 | 0.019 | | 2.36E-08 | 1.50E-04 | 31.221 | #N/A | | #N/A | | #N/A | |
| rs750597 | | A | | T | | -0.096 | 0.015 | | 6.64E-11 | 2.10E-04 | 42.826 | #N/A | | #N/A | | #N/A | |
| rs137943060 | | A | | G | | 0.331 | 0.051 | | 5.42E-11 | 2.10E-04 | 42.96 | 0.025 | | 0.048 | | 0.604 | |
| rs12906835 | | G | | A | | -0.115 | 0.014 | | 5.26E-16 | 3.20E-04 | 65.701 | -0.006 | | 0.019 | | 0.729 | |
| rs191156695 | | T | | C | | -0.262 | 0.042 | | 2.96E-10 | 1.90E-04 | 39.665 | #N/A | | #N/A | | #N/A | |
| rs73015021 | | G | | A | | -0.165 | 0.023 | | 1.21E-12 | 2.50E-04 | 50.642 | -0.008 | | 0.022 | | 0.734 | |
| rs7412 | | T | | C | | -0.211 | 0.031 | | 6.05E-12 | 2.30E-04 | 47.456 | -0.467 | | 0.031 | | 6E-53 | |
| rs9980618 | | T | | C | | 0.123 | 0.020 | | 9.60E-10 | 1.80E-04 | 37.264 | -0.026 | | 0.022 | | 0.231 | |
|  | |  | |  | | **Ischemic Stroke** | | | | | | **Alzheimer's Disease** | | | | | |
| **SNP** | | **effect allele** | | **other allele** | | **Beta.**  **exposure** | **Se.**  **exposure** | | **Pval.**  **exposure** | **R2** | **F** | **Beta.**  **outcome** | | **Se.**  **outcome** | | **Pval.**  **outcome** | |
| rs17035646 | | A | | G | | 0.054 | 0.009 | | 1.34E-09 | 8.40E-05 | 37.099 | -6E-04 | | 0.015 | | 0.97 | |
| rs2842873 | | T | | C | | -0.057 | 0.009 | | 4.86E-11 | 9.80E-05 | 43.227 | -0.024 | | 0.015 | | 0.104 | |
| rs6847935 | | T | | A | | 0.078 | 0.010 | | 3.50E-16 | 1.50E-04 | 66.694 | #N/A | | #N/A | | #N/A | |
| rs6825454 | | C | | T | | 0.056 | 0.009 | | 7.43E-10 | 8.50E-05 | 37.582 | 0.0018 | | 0.017 | | 0.916 | |
| rs11957829 | | G | | A | | -0.072 | 0.012 | | 7.51E-09 | 7.60E-05 | 33.621 | -0.02 | | 0.019 | | 0.293 | |
| rs4959130 | | A | | G | | 0.083 | 0.014 | | 2.83E-09 | 8.00E-05 | 35.317 | -0.023 | | 0.021 | | 0.274 | |
| rs42039 | | T | | C | | -0.066 | 0.011 | | 6.55E-09 | 7.70E-05 | 33.701 | -0.056 | | 0.017 | | 7E-04 | |
| rs2107595 | | A | | G | | 0.076 | 0.010 | | 9.25E-14 | 1.30E-04 | 55.371 | 0.0004 | | 0.019 | | 0.984 | |
| rs7859727 | | T | | C | | 0.051 | 0.008 | | 1.05E-09 | 8.50E-05 | 37.443 | 0.0327 | | 0.015 | | 0.031 | |
| rs2005108 | | T | | C | | 0.080 | 0.015 | | 3.33E-08 | 6.90E-05 | 30.44 | -0.013 | | 0.022 | | 0.548 | |
| rs35436 | | T | | C | | -0.050 | 0.009 | | 3.21E-08 | 7.00E-05 | 30.933 | -0.009 | | 0.015 | | 0.542 | |
| rs3184504 | | C | | T | | -0.075 | 0.010 | | 2.17E-14 | 1.30E-04 | 58.725 | 0.0255 | | 0.014 | | 0.075 | |
| rs7304841 | | C | | A | | -0.048 | 0.009 | | 4.93E-08 | 6.70E-05 | 29.574 | 0.0179 | | 0.016 | | 0.247 | |
| rs9526212 | | G | | A | | 0.062 | 0.010 | | 9.19E-10 | 8.40E-05 | 37.077 | -0.013 | | 0.017 | | 0.457 | |
| rs4932370 | | A | | G | | 0.052 | 0.009 | | 2.88E-08 | 6.90E-05 | 30.484 | 0.0034 | | 0.016 | | 0.832 | |
| rs12445022 | | A | | G | | 0.061 | 0.010 | | 1.28E-10 | 9.30E-05 | 41.095 | 0.0317 | | 0.015 | | 0.037 | |
| rs9909858 | | C | | T | | 0.089 | 0.016 | | 3.63E-08 | 6.90E-05 | 30.386 | -0.036 | | 0.03 | | 0.225 | |
| rs1053007 | | G | | A | | 0.048 | 0.009 | | 3.58E-08 | 6.90E-05 | 30.313 | #N/A | | #N/A | | #N/A | |
|  |  | |  | | **Large-artery Atherosclerotic Stroke** | | | | | | | **Alzheimer's Disease** | | | | |  |
| **SNP** | **effect allele** | | **other allele** | | **Beta** | | **Se** | **Pval** | | **R2** | **F** | **Beta** | **se** | | **pval** | |  |
| rs12124533 | T | | C | | 0.153 | | 0.027 | 1.22E-08 | | 2.20E-04 | 32.549 | 0.0359 | 0.017 | | 0.032 | |  |
| rs17612742 | C | | T | | 0.176 | | 0.026 | 1.46E-11 | | 3.00E-04 | 45.471 | -0.028 | 0.021 | | 0.177 | |  |
| rs2107595 | A | | G | | 0.187 | | 0.024 | 3.65E-15 | | 4.10E-04 | 61.8 | 0.0004 | 0.019 | | 0.984 | |  |
| rs72985562 | G | | T | | 0.245 | | 0.044 | 2.42E-08 | | 2.10E-04 | 31.12 | 0.0018 | 0.028 | | 0.948 | |  |
|  |  | |  | | **Cardioembolic Stroke** | | | | | | | **Alzheimer's Disease** | | | | |  |
| **SNP** | **effect allele** | | **other allele** | | **Beta** | | **se** | **pval** | | **R2** | **F** | **Beta** | **se** | | **pval** | |  |
| rs13143308 | G | | T | | -0.280 | | 0.019 | 1.86E-47 | | 9.90E-04 | 210.17 | 0.0189 | 0.017 | | 0.276 | |  |
| rs1906613 | A | | G | | 0.102 | | 0.018 | 1.83E-08 | | 1.50E-04 | 31.695 | -0.019 | 0.015 | | 0.199 | |  |
| rs55884259 | A | | G | | -0.110 | | 0.019 | 3.87E-09 | | 1.60E-04 | 34.721 | 0.0152 | 0.016 | | 0.331 | |  |
| rs12932445 | C | | T | | 0.184 | | 0.021 | 6.86E-18 | | 3.50E-04 | 74.542 | -0.03 | 0.019 | | 0.124 | |  |

#effect_allele= effect alleles; other_allele=minor allele; beta.exposure=effect size of SNP on exposure; se.exposure =standard error for exposure; pval.exposure =p-value for exposure; beta.outcome=effect size of SNP on outcome; se.outcome =standard error for outcome; pval.outcome =p-value for outcome; R2= the proportion of variance in the exposure explained by the genetic variants; F= F-statistic,F-statistic greater than or equal to 10 indicates a relatively low risk of weak instrument bias in MR analysis.#N/A stands for actual SNP information.
